# Supplementary material for: Fusobacterium nucleatum interacts with cancer-associated fibroblasts to promote colorectal cancer
Source: EMBO J. 2025 Aug 22;44(19):5375–93. doi: 10.1038/s44318-025-00542-w (PMC12488894; doi:10.1038/s44318-025-00542-w)
Supplement: Supplementary file 1 — Appendix [file 44318_2025_542_MOESM1_ESM.pdf]

APPENDIX for

Fusobacterium nucleatum interacts with cancer-associated fibroblasts to promote colorectal cancer

Jessica Karta[1,10], Marianne Meyers[1,10], Fabien Rodriguez[1], Eric Koncina[1], Cedric Gilson[1], Eliane Klein[1], Monica Gabola[1], Mohaned Benzarti[1], Pau Pérez Escriva[1], Jose Alberto Molina Tijeras[1], Catarina Correia Tavares Bernardino[1], Falk Ponath[2], Anais Carpentier[3], Mònica Aguilera Pujabet [4], Maryse Schmoetten[1], Mina Tsenkova[1], Perla Saoud[1], Anthoula Gaigneaux[1], Dominik Ternes[1], Lidia Alonso[4], Nikolaus Zügel[5], Eric Willemssen[6], Philippe Koppes[6], Daniel Léonard[6], Luis Perez Casanova[3], Serge Haan[1], Michel Mittelbronn[1,3], Johannes Meiser[7], Vitaly I Pozdeev[1], Jörg Vogel[2,8], Paolo G Nuciforo[4], Paul Wilmes[1,9] & Elisabeth Letellier[1✉]

[1] Department of Life Sciences and Medicine (DLSM), Faculty of Science, Technology and Medicine, University of Luxembourg, Esch-sur-Alzette, Luxembourg

[2] Institute of Molecular Infection Biology, University of Würzburg, Würzburg, Germany

[3] Laboratoire National de Santé (LNS), National Center of Pathology (NCP), Dudelange, Luxembourg

[4] Molecular Oncology Group, Vall d'Hebron Institute of Oncology, Barcelona, Spain

[5] Centre Hospitalier Emile Mayrisch, Esch-sur-Alzette, Luxembourg

[6] Groupe Chirurgical Zitha, Hôpitaux Robert Schuman, Luxembourg, Luxembourg

[7] Department of Cancer Research (DOCR), Luxembourg Institute of Health, Luxembourg, Luxembourg

[8] Helmholtz Institute for RNA-based Infection Research (HIRI), Helmholtz Centre for Infection Research (HZI), D-97080 Würzburg, Germany

[9] Luxembourg Centre for Systems Biomedicine, University of Luxembourg, Esch-sur-Alzette, Luxembourg

[10] These authors contributed equally: Jessica Karta, Marianne Meyers.

✉ E-mail: elisabeth.letellier@uni.lu

Table of contents

Appendix Figure S1 1

Appendix Figure S2 2

Appendix Figure S3 4

Appendix Figure S4 5

Appendix Table S1 6

Appendix Table S2 7

Appendix Table S3 8

Appendix Table S4 9

Appendix Table S5 10

Appendix Figure S1

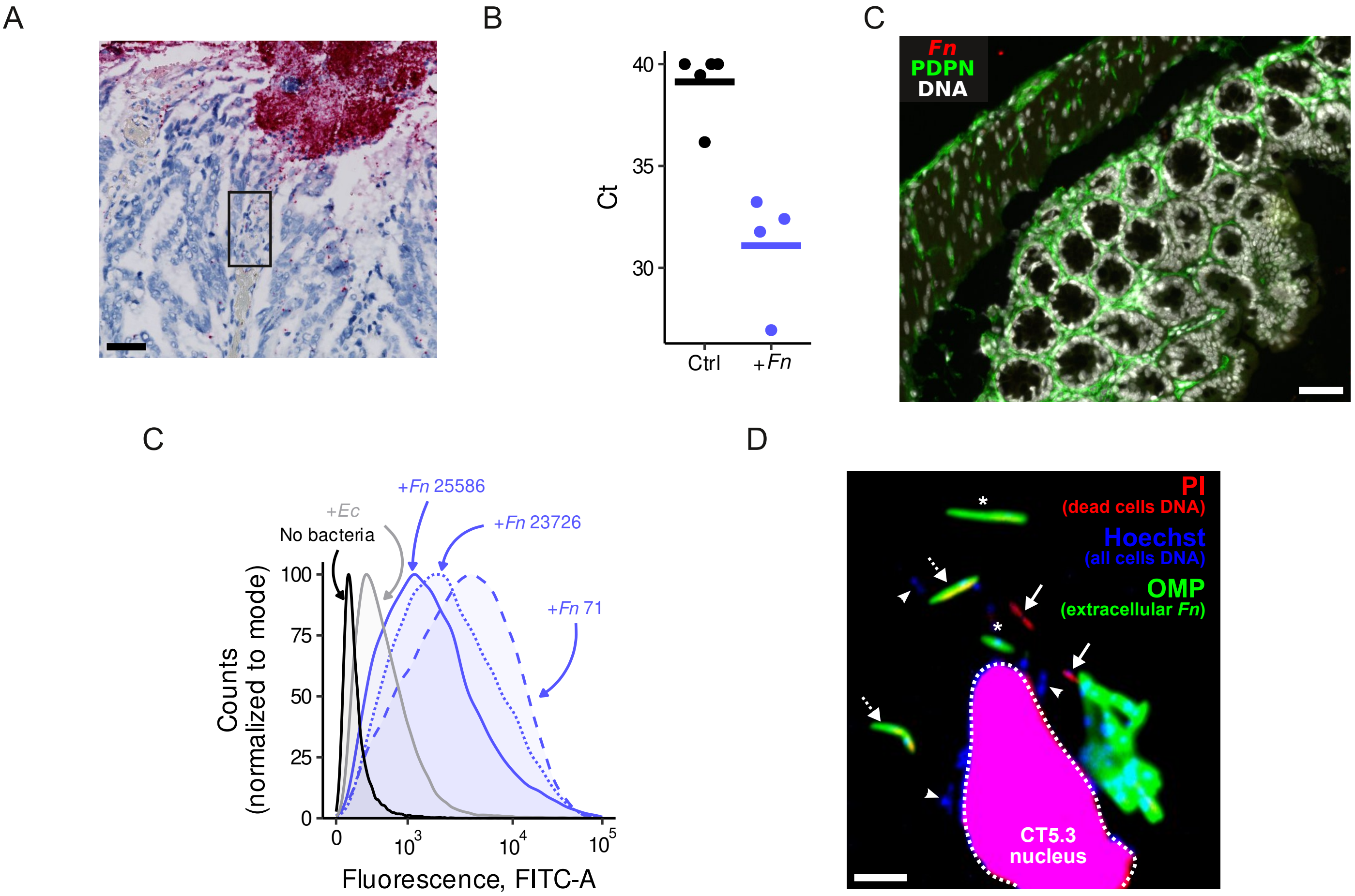

Appendix Figure S1

A. Original *in situ* hybridization staining of human CRC tissue prior to αSMA counter staining from Figure 1B for improved visualization of tissue structure. The same field of view and outlined region of interest (black rectangle) are shown. Scale bar = 100 µm. **B**. Cycle threshold value (Ct) of *Fn* 71 qPCR in stool from the germ free *CDX2-CreER*^{T2}*Apc*$^{fl/fl}$ experiment, corresponding to main Figure 1D-E, collected at end point (n = 5 PBS- and 4 *Fn*-treated mice). **C.** Representative immunofluorescent image showing no colocalization of *Fn* (red, stained with an OMP *Fn* specific antibody) with the stromal marker PDPN (green), and DAPI (grey) in the colon from a PBS-gavaged *CDX2-CreER*^{T2}*Apc*$^{fl/fl}$ mouse (as a control for the main Figure 1E). Scale bar = 50 µm. **D.** Example flow cytometry histogram of bound or invading CFSE-stained-bacteria (*Fn* 25586, *Fn* 23726, *Fn* 71 or *Ec*, MOI 50, 4-hour co-culture) in CT5.3 CAFs, corresponding to main Figure 1H. **E.** Viability staining of a representative CT5.3 CAF co-cultured with MOI 50 *Fn* 71 for four hours (n = 1 independent experiment). Live extracellular *Fn* are indicated by the asterisk (*) and are OMP$^+$ as well as Hoechst$^+$. Dead extracellular *Fn* are indicated by the dashed arrow and are OMP$^+$, PI$^+$ and Hoechst$^+$. Dead intracellular bacteria are indicated by a solid arrow and are Hoechst$^+$ and PI$^+$. And finally, live intracellular bacteria, indicated by the arrowhead, are only Hoechst$^+$. The dashed line surrounding the pink area encloses the nuclei of the resident CAF. Scale bar = 5 µm. The horizontal line in B shows the mean.

Appendix Figure S2

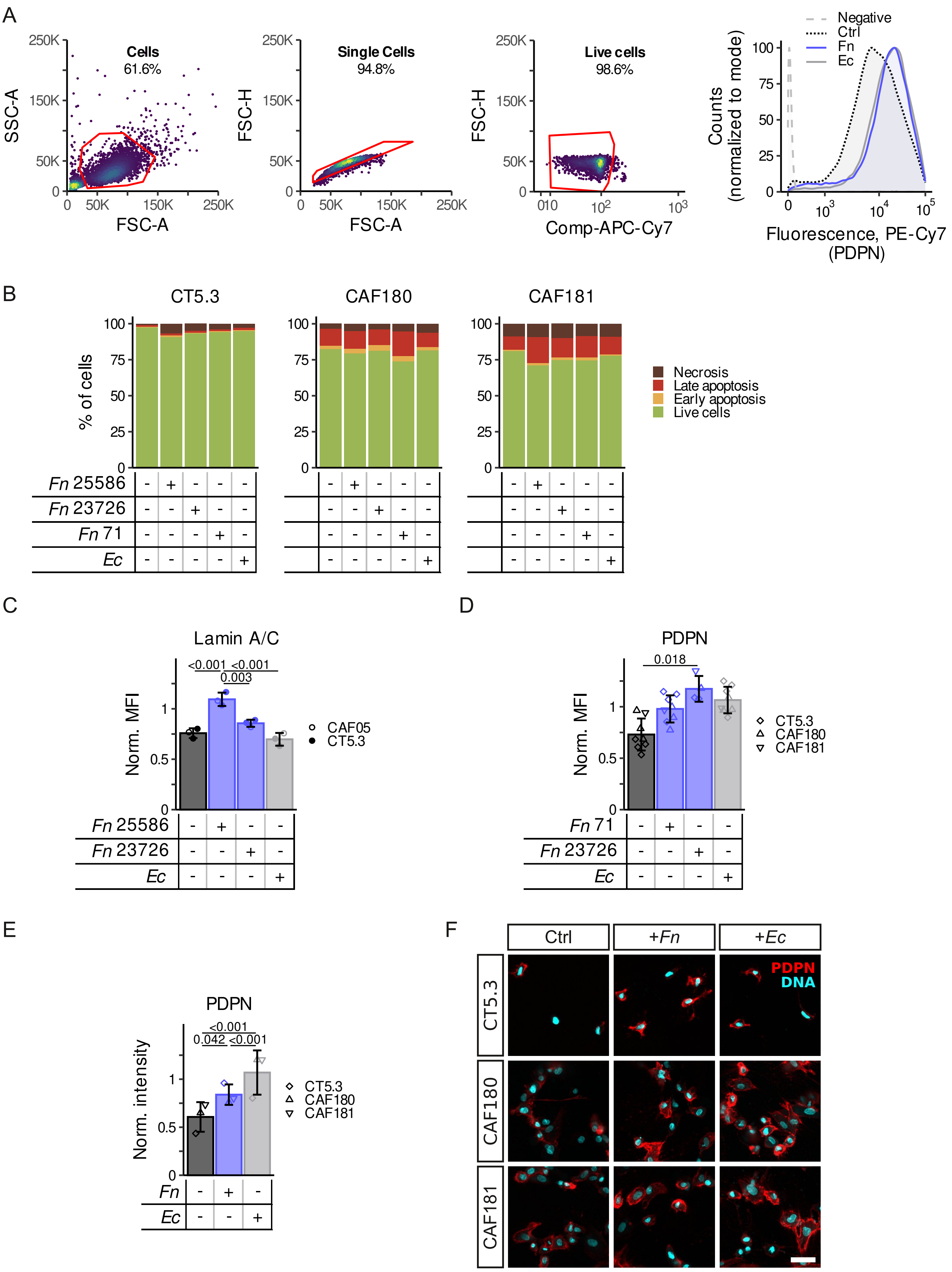

Appendix Figure S2

A. Flow cytometry gating strategies of the CAF markers, here podoplanin (PDPN). **B.** Cell viability of CT5.3 CAFs and the patient derived CAF180 and CAF181 CAFs treated with *Fn* 25586, *Fn* 23726, *Fn* 71 or *Ec* for four hours at an MOI of 50, determined by the Annexin IV assay via flow cytometry (n = 3 independent experiments). **C.** Expression of Lamin A/C 24 hours after a two-hour treatment with either *Fn* 25586, *Fn* 23726 or *Ec* (MOI 500) as measured by flow cytometry (Each data point represents a technical replicate from n = 2 independent experiments). **D.** Expression of PDPN in CT5.3 CAFs 24 hours after a four-hour treatment with either *Fn* 25586, *Fn* 23726 or *Ec* (MOI 50) as measured by flow cytometry (n = 3 independent experiments). **E.** Normalized mean fluorescent intensity of PDPN in the cytoplasm of CT5.3 cells treated for four hours with *Fn* 71 or *Ec* (MOI 50, n = 1 independent experiment). **F.** Representative immunofluorescent images of PDPN (red) staining, quantified in E, counter stained with DAPI (blue). Scale bar = 20 µm. PDPN = podoplanin. The bar chart and error bars show the mean ± SD and statistically significant differences were determined using a nested ANOVA followed by Tukey's HSD post-hoc test.

Appendix Figure S3

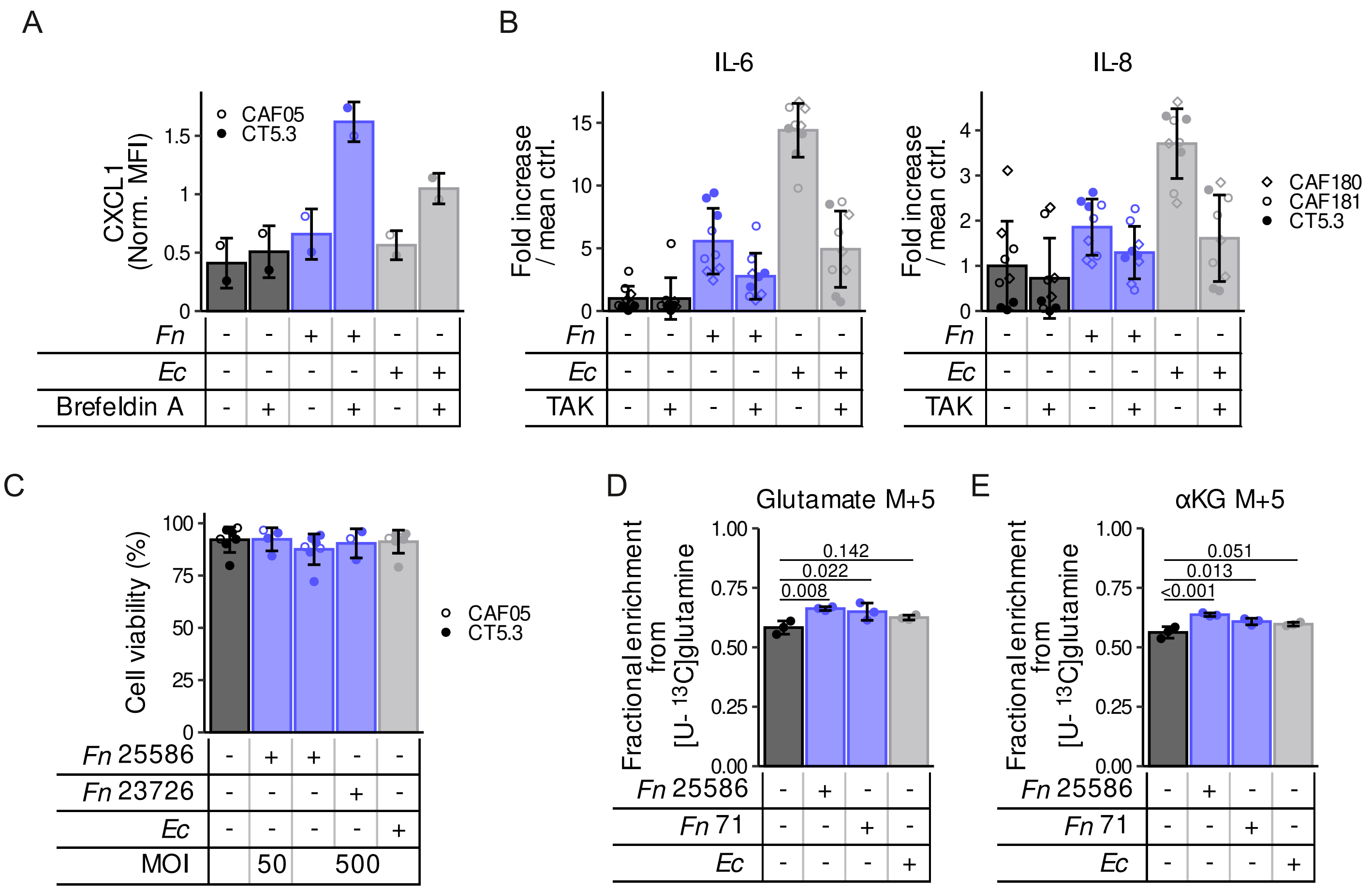

Appendix Figure S3

A. CXCL1 quantified by flow cytometry after 24 hours in CT5.3 and CAF05 CAFs co-incubated with *Fn* 25586 or *Ec* for two-hours (MOI 500), and upon treatment with Brefeldin A (n = 1 independent experiments for CAF05 and CT5.3). **B.** ELISA results for IL-6 and IL-8 replotted from the main Figure 4C and D, with the additional conditions of TAK-242, a TLR4 inhibitor, pre-treatment (n = 3 independent experiments with 3 independent cell lines). **C**. Cell viability of CAFs treated with *Fn* 25586, *Fn* 23726 or *Ec* for two-hours (MOI 500). CAF05 and CT5.3 cell viabilities were assessed by live/dead staining by flow cytometry (summarized from 7 independent experiments with 4-7 replicates per condition). **D-E.** Fractional enrichment in M+5 glutamate (**D**) and M+5 alpha-ketoglutarate (**E**) from [U-^{13}C]glutamine after a four-hour co-culture of CT5.3 with either *Fn* 71, *Fn* 25586, *Ec* or PBS control (MOI 50), followed by a 24-hour incubation with [U^{13}C]glutamine. The bar chart and error bars show the mean ± SD and statistically significant differences were determined using a nested ANOVA followed by Tukey's HSD post-hoc test.

Appendix Figure S4

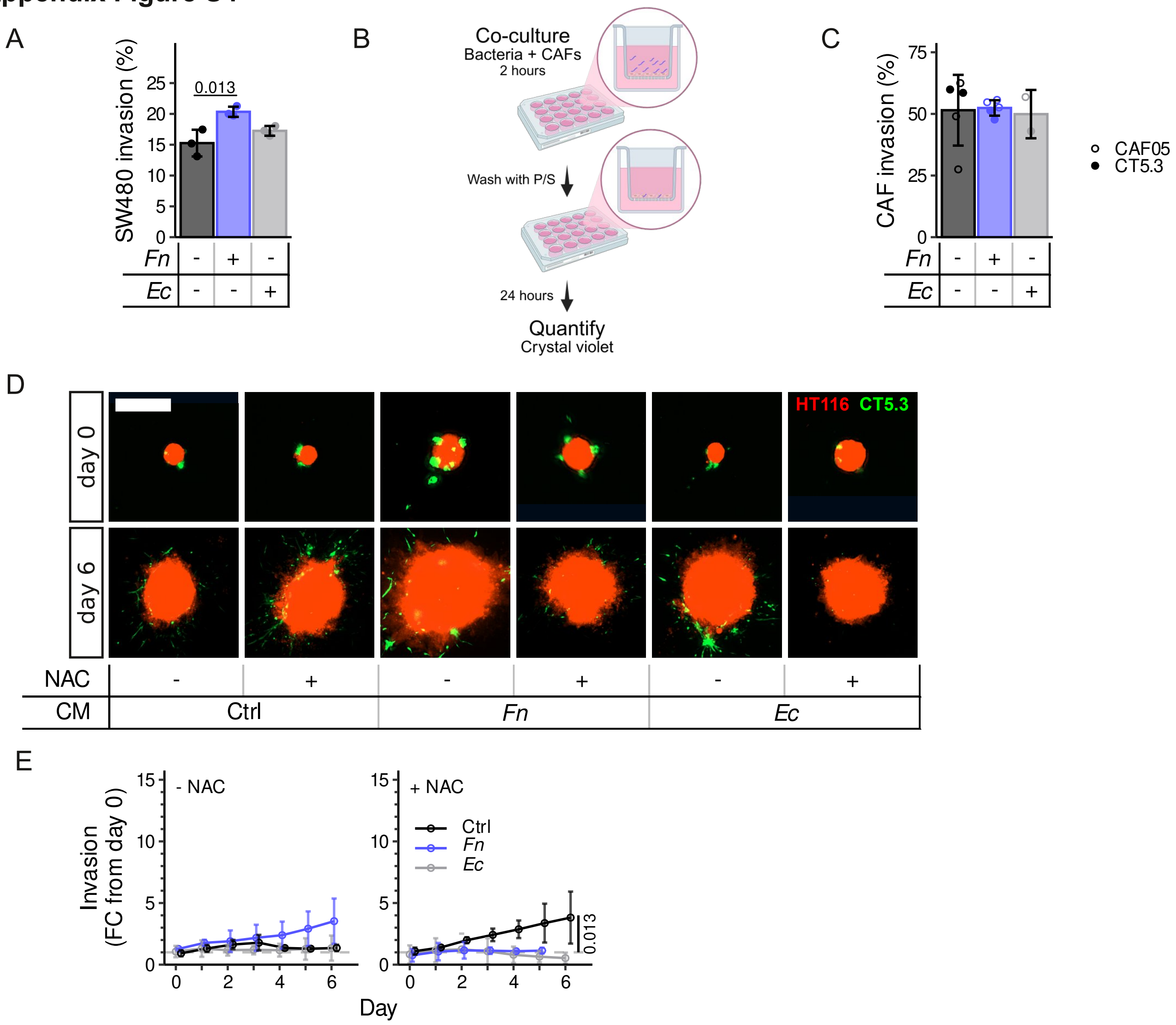

Appendix Figure S4

A. Percentage of SW480 cells that invaded upon co-culture with four-hour non- and bacteria (*Fn* 71 and *Ec*)-treated CT5.3 CAFs (MOI 50, n = 3 biological replicates). **B**. Schematic representation of the Transwell assay for CAF invasion. **C.** Percentage of CAFs that migrated after 24 hours post two-hour co-culture with *Fn* 25586 or *Ec* (MOI 500, n = 3 and n = 2 independent experiments for CAF05 and CT5.3, respectively). **D**. Representative pictures of the *in vitro* complex 3D co-culture experiment. Overlay shows mCherry HCT116 cells in red and GFP CT5.3 in green. Pictures represent 3 independent experiments with a minimum of 2 technical replicates included per experiment. Scale bar = 1 mm. **E**. Invasion of the 3D complex spheroid models as quantified by CT5.3 (GFP) outgrowth from the core normalized to day 0 over the total span of the experiment, 6 days (n = 3 independent experiments, a minimum of 2 technical replicated included per experiment).The data points and error bars show the mean ± SD and statistically significant differences were determined using pairwise 2-way repeated measure ANOVAs (time × treatment). The interaction term p-values were adjusted using Holm’s method.

Appendix Table S1 – List of cell lines

Cell line	Supplier
CT5.3 hTERT, Immortalized Human Colonic Tumor Fibroblast	Cancer Research Institute Ghent
CAF05, Commercial Human Colorectal Tumor Fibroblast	Neuromics
HCT116	ATCC
HT-29	ATCC
SW480	ATCC
T4/CAF4	Primary derived cell line from in house CRC patient cohort
CAF12	
CAF16	
CAF19	
CAF20	
CAF22	
CAF32	
CAF41	
CAF42	
CAF180	
CAF181	

Appendix Table S2 – List of bacterial strains

**Bacteria**	**Supplier**
Fusobacterium nucleatum subsp. *Nucleatum* 25586	ATCC
Fusobacterium nucleatum subsp. *Nucleatum* 23726	ATCC
Fusobacterium nucleatum subsp. *Animalis* 7_1	Virginia Tech
Fap2 mutant *Fusobacterium nucleatum* subsp. *Nucleatum* 23726	Jorg Vogel
FadA mutant *Fusobacterium nucleatum* subsp. *Nucleatum* 23726	Jorg Vogel
Escherichia coli K-12 MG1655	ESB group, LCSB

Appendix Table S3 – Vectors and oligonucleotides used to generate *FnΔfap2*

Strain ID	Original strain	Description	Origin
FPS-649	*Δfap2 Fusobacterium nucleatum* subsp. *nucleatum* ATCC 23726	deletion of full-length *fap2*; deletion carried according to protocol described in methods and PMID: 36161895	This study
FPS-189	*ΔfadA Fusobacterium nucleatum* subsp. *nucleatum* ATCC 23726	deletion of full-length *fadA*	PMID: 36161895

Strain ID	Description	Origin
pVoPo-04	base vector; markerless gene deletion vector for *F. nucleatum*	PMID: 36161895
pFP468	pVoPo-04-fap2; gene deletion vector for *fap2*	this study

Oligo ID	Sequence	Description
JVO-20976	`aatttttacttatattttatcgatcGTAGTGATAAAGATGCTGGAAAAAATAC`	Gibson Assembly; forward *fap2* homolog arm upstream
JVO-20977	`taagcctttttaaATAATTTCCCCCTTTTTATTTTATATTTTAAATTATAC`	Gibson Assembly; reverse *fap2* homolog arm upstream
JVO-20978	`gggggaaattatTTAAAAAGGCTTAAATTTACAATTAACATAATTAAG`	Gibson Assembly; forward *fap2* homolog arm downstream
JVO-20979	`ggagttctgaggtcattactggagcggccGCTTGTGTAGAATCATTTCCTGTTAAC`	Gibson Assembly; reverse *fap2* homolog arm downstream
JVO-20156	`ATAACAGGTGGAATGACATCAGCACCAGG`	Verification; forward *fap2* deletion
JVO-20157	`AATGCATCCCCTCCCAACATTAAAAACAGTATTGC`	Verification; reverse *fap2* deletion

Appendix Table S4 – List of primers

Primer	Sequence
YWAHZ - F	5'-ACTTTTGGTACATTGTGGCTTCAA-3'
YWAHZ - R	5'-CCGCCAGGACAAACCAGTAT-3'
HPRT1 - F	5'-TGGACAGGACTGAACGTCTT-3'
HPRT1 - R	5'-GAGCACACAGAGGGCTACAA-3'
ACTA2 - F	5'-CTATGCCTCTGGACGCACAACT-3'
ACTA2 - R	5'-CAGATCCAGACGCATGATGGCA-3'
IL6 - F	5'-AGACAGCCACTCACCTCTTCAG-3'
IL6 - R	5'-TTCTGCCAGTGCCTCTTTGCTG-3'

Appendix Table S5 – Exact p-values for all figures

Figure 1H

Comparison	p-value / adj. p-value Exact	Formatted
**CAF**		
Fn (MOI 50) vs Ctrl	7.209e-01	0.721
Fn (MOI 500) vs Ctrl	<1e-15	<0.001
Ec vs Ctrl	9.124e-01	0.912
Fn (MOI 500) vs *Fn* (MOI 50)	8.327e-15	<0.001
Ec vs *Fn* (MOI 50)	9.594e-01	0.959
Ec vs *Fn* (MOI 500)	<1e-15	<0.001
**Tumor**		
Fn (MOI 50) vs Ctrl	9.926e-01	0.993
Fn (MOI 500) vs Ctrl	6.891e-05	<0.001
Ec vs Ctrl	9.999e-01	>0.999
Fn (MOI 500) vs *Fn* (MOI 50)	2.933e-04	<0.001
Ec vs *Fn* (MOI 50)	9.967e-01	0.997
Ec vs *Fn* (MOI 500)	8.370e-05	<0.001

Figure 1I

Comparison	p-value / adj. p-value Exact	Formatted
Fn 25586 vs Ctrl	7.520e-11	<0.001
Fn 23726 vs Ctrl	<1e-15	<0.001
Fn 71 vs Ctrl	<1e-15	<0.001
Ec vs Ctrl	6.675e-01	0.667
Fn 23726 vs *Fn* 25586	3.431e-01	0.343
Fn 71 vs *Fn* 25586	5.683e-11	<0.001
Ec vs *Fn* 25586	4.910e-07	<0.001
Fn 71 vs *Fn* 23726	8.553e-06	<0.001
Ec vs *Fn* 23726	1.836e-12	<0.001
Ec vs *Fn* 71	<1e-15	<0.001

Figure 2D

Comparison	p-value / adj. p-value Exact	Formatted
+*Fn* vs +*FnΔfap2*	5.772e-05	<0.001
+*Fn* vs +*FnΔfadA*	9.998e-01	>0.999
+*Fn* vs +*Ec*	2.858e-11	<0.001
Ctrl vs +*Fn*	<1e-15	<0.001

Figure 2E

Comparison	p-value / adj. p-value Exact	Formatted
Fn vs Ctrl	<1e-15	<0.001
Fn + GalNAc vs Ctrl	<1e-15	<0.001
Fn + GalNAc vs *Fn*	<1e-15	<0.001

Figure 3B

Comparison	p-value / adj. p-value Exact	Formatted
**iCAF markers**		
CXCL1 : Fn^{hi} vs Fn^{lo}	2.520e-10	<0.001
CXCL2 : Fn^{hi} vs Fn^{lo}	1.850e-10	<0.001
CXCL3 : Fn^{hi} vs Fn^{lo}	3.199e-08	<0.001
IL6 : Fn^{hi} vs Fn^{lo}	6.420e-08	<0.001
MMP1 : Fn^{hi} vs Fn^{lo}	7.624e-09	<0.001
**myCAF markers**		
MMP11 : Fn^{hi} vs Fn^{lo}	1.245e-03	0.001
MYH11 : Fn^{hi} vs Fn^{lo}	1.245e-03	0.001
MYL9 : Fn^{hi} vs Fn^{lo}	1.074e-02	0.011
RGS5 : Fn^{hi} vs Fn^{lo}	1.245e-03	0.001
TPM2 : Fn^{hi} vs Fn^{lo}	1.540e-02	0.015

Figure 3D

Comparison	p-value / adj. p-value Exact	Formatted
**PDGFRα**		
Fn vs Ctrl	9.867e-02	0.099
Ec vs Ctrl	1.000e+00	>0.999
Ec vs *Fn*	9.966e-02	0.100
**Lamin A/C**		
Fn vs Ctrl	1.293e-13	<0.001
Ec vs Ctrl	7.907e-01	0.791
Ec vs *Fn*	1.110e-15	<0.001
**αSMA**		
Fn vs Ctrl	1.465e-01	0.146
Ec vs Ctrl	2.536e-01	0.254
Ec vs *Fn*	9.872e-01	0.987
**PDGFRβ**		
Fn vs Ctrl	7.053e-10	<0.001
Ec vs Ctrl	7.899e-08	<0.001
Ec vs *Fn*	7.483e-01	0.748

Figure 3H

Comparison	p-value / adj. p-value Exact	Formatted
**iCAF**		
Fn^{hi} vs Fn^{lo} (Neither CMS4 nor CRIS-B)	3.120e-09	<0.001
Fn^{hi} vs Fn^{lo} (CMS4 or CRIS-B)	6.110e-03	0.006
**myCAF**		
Fn^{hi} vs Fn^{lo} (Neither CMS4 nor CRIS-B)	1.220e-01	0.122
Fn^{hi} vs Fn^{lo} (CMS4 or CRIS-B)	4.600e-03	0.005

Figure 4B

Comparison	p-value / adj. p-value Exact	Formatted
Fn vs Ctrl	3.100e-05	<0.001
Ec vs Ctrl	2.190e-04	<0.001
Ec vs *Fn*	2.220e-02	0.022

Figure 4C

Comparison	p-value / adj. p-value Exact	Formatted
Fn vs Ctrl	2.242e-02	0.022
Ec vs Ctrl	7.885e-08	<0.001
Ec vs *Fn*	9.263e-03	0.009

Figure 4D

Comparison	p-value / adj. p-value Exact	Formatted
Fn vs Ctrl	1.481e-01	0.148
Ec vs Ctrl	4.610e-04	<0.001
Ec vs *Fn*	1.308e-01	0.131

Figure 4E

Comparison	p-value / adj. p-value Exact	Formatted
iCAF vs myCAF	<1e-15	<0.001

Figure 4F

Comparison	p-value / adj. p-value Exact	Formatted
Fn^{hi} vs Fn^{lo}	1.080e-03	0.001

Figure 4G

	p-value / adj. p-value	
Comparison	Exact	Formatted
**DCFDA**		
Fn vs Ctrl	2.169e-02	0.022
Ec vs Ctrl	8.751e-01	0.875
Ec vs *Fn*	3.941e-01	0.394
**MitoSOX**		
Fn vs Ctrl	2.817e-06	<0.001
Ec vs Ctrl	1.585e-01	0.159
Ec vs *Fn*	8.902e-02	0.089

Figure 5E

	p-value / adj. p-value	
Comparison	Exact	Formatted
Ctrl vs Ctrl (tumor only)	5.010e-07	<0.001
Fn (MOI 50) vs Ctrl (tumor only)	3.796e-08	<0.001
Fn (MOI 500) vs Ctrl (tumor only)	5.662e-15	<0.001
Ec (MOI 500) vs Ctrl (tumor only)	6.456e-06	<0.001
Fn (MOI 50) vs Ctrl	9.661e-01	0.966
Fn (MOI 500) vs Ctrl	1.830e-02	0.018
Ec (MOI 500) vs Ctrl	9.930e-01	0.993
Fn (MOI 500) vs *Fn* (MOI 50)	1.939e-01	0.194
Ec (MOI 500) vs *Fn* (MOI 50)	9.998e-01	>0.999
Ec (MOI 500) vs *Fn* (MOI 500)	1.541e-01	0.154

Figure 5H

	p-value / adj. p-value	
Comparison	Exact	Formatted
**- NAC**		
Fn vs Ctrl (day × CM interaction term)	2.593e-05	<0.001
Ec vs Ctrl (day × CM interaction term)	1.000e+00	>0.999
**+ NAC**		
Fn vs Ctrl (day × CM interaction term)	1.000e+00	>0.999
Ec vs Ctrl (day × CM interaction term)	7.571e-01	0.757

Figure 5I

	p-value / adj. p-value	
Comparison	Exact	Formatted
Fn vs Ctrl	1.224e-04	<0.001
Fn + NAC vs Fn	2.271e-04	<0.001
Ec vs Fn	2.028e-04	<0.001
Ec + NAC vs Fn	1.541e-03	0.002

Figure 6B

	p-value / adj. p-value	
Comparison	Exact	Formatted
Ctrl vs Fn	1.598e-02	0.016
Ctrl vs Ec	8.146e-01	0.815
Fn vs Ec	6.098e-03	0.006

Appendix Figure S2C

	p-value / adj. p-value	
Comparison	Exact	Formatted
Fn 25586 vs Ctrl	5.876e-06	<0.001
Fn 23726 vs Ctrl	4.880e-01	0.488
Ec vs Ctrl	8.428e-01	0.843
Fn 23726 vs *Fn* 25586	3.200e-03	0.003
Ec vs *Fn* 25586	3.584e-08	<0.001
Ec vs *Fn* 23726	1.118e-01	0.112

Appendix Figure S2D

	p-value / adj. p-value	
Comparison	Exact	Formatted
Fn 71 vs Ctrl	7.850e-01	0.785
Fn 23726 vs Ctrl	1.773e-02	0.018
Ec vs Ctrl	8.229e-02	0.082
Fn 23726 vs *Fn* 71	9.844e-02	0.098
Ec vs *Fn* 71	4.724e-01	0.472
Ec vs *Fn* 23726	5.488e-01	0.549

Appendix Figure S2E

	p-value / adj. p-value	
Comparison	Exact	Formatted
Ec vs Ctrl	<1e-15	<0.001
Fn vs Ctrl	4.196e-02	0.042
Ec vs *Fn*	1.155e-13	<0.001

Appendix Figure S3D

	p-value / adj. p-value	
Comparison	Exact	Formatted
Ctrl vs Fn 25586	8.155e-03	0.008
Ctrl vs Fn 71	2.190e-02	0.022
Ctrl vs *Ec*	1.423e-01	0.142

Appendix Figure S3E

	p-value / adj. p-value	
Comparison	Exact	Formatted
Ctrl vs Fn 25586	6.594e-04	<0.001
Ctrl vs Fn 71	1.349e-02	0.013
Ctrl vs *Ec*	5.139e-02	0.051

Appendix Figure S4A

	p-value / adj. p-value	
Comparison	Exact	Formatted
Ctrl vs *Fn*	1.340e-02	0.013
Ctrl vs *Ec*	2.660e-01	0.266
Fn vs *Ec*	1.080e-01	0.108

Appendix Figure S4C

	p-value / adj. p-value	
Comparison	Exact	Formatted
Fn vs Ctrl	9.636e-01	0.964
Ec vs Ctrl	9.792e-01	0.979
Ec vs *Fn*	9.207e-01	0.921

Appendix Figure S4E

	p-value / adj. p-value	
Comparison	Exact	Formatted
**- NAC**		
Fn vs Ctrl (day × CM interaction term)	1.570e-01	0.157
Ec vs Ctrl (day × CM interaction term)	8.886e-01	0.889
**+ NAC**		
Fn vs Ctrl (day × CM interaction term)	1.570e-01	0.157
Ec vs Ctrl (day × CM interaction term)	1.322e-02	0.013
