# Supplementary figures and images for: Fusobacterium nucleatum interacts with cancer-associated fibroblasts to promote colorectal cancer
Source: EMBO J. 2025 Aug 22;44(19):5375–93. doi: 10.1038/s44318-025-00542-w (PMC12488894; doi:10.1038/s44318-025-00542-w)

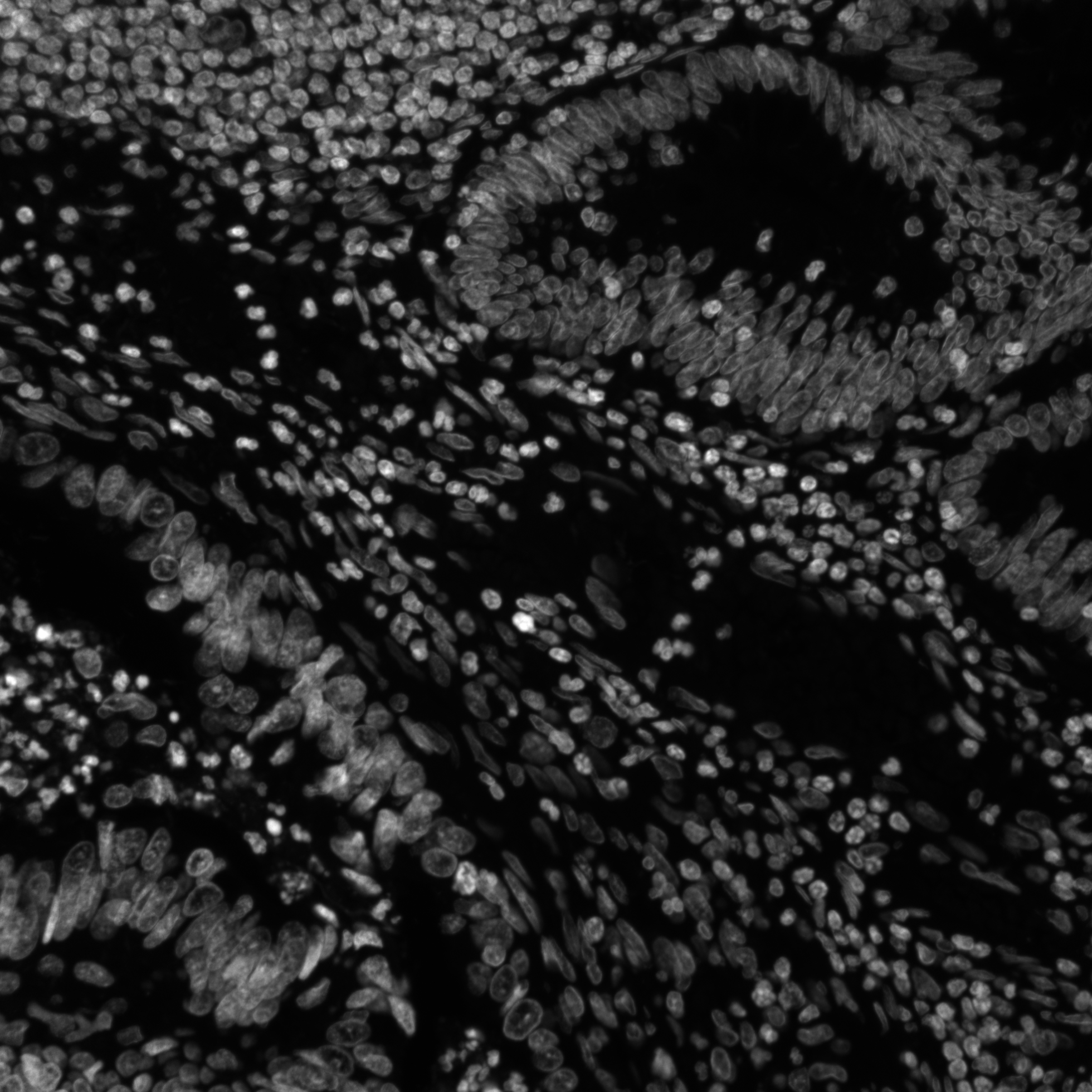

Supplement: Supplementary file 3 — Source data Fig. 1 [file 44318_2025_542_MOESM3_ESM.zip › Figure 1/1F/Fn_DAPI.tif]

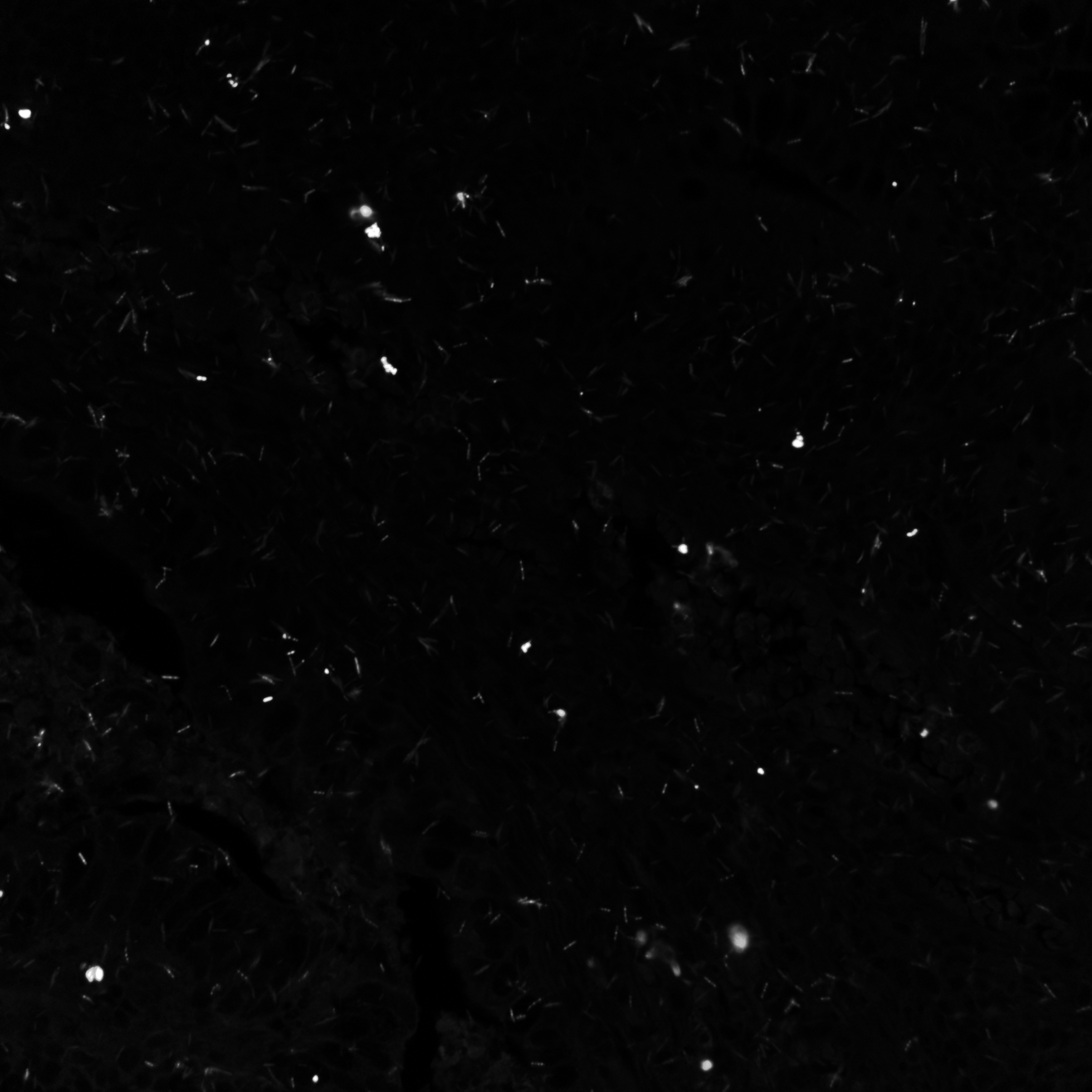

Supplement: Supplementary file 3 — Source data Fig. 1 [file 44318_2025_542_MOESM3_ESM.zip › Figure 1/1F/Fn_CY5.tif]

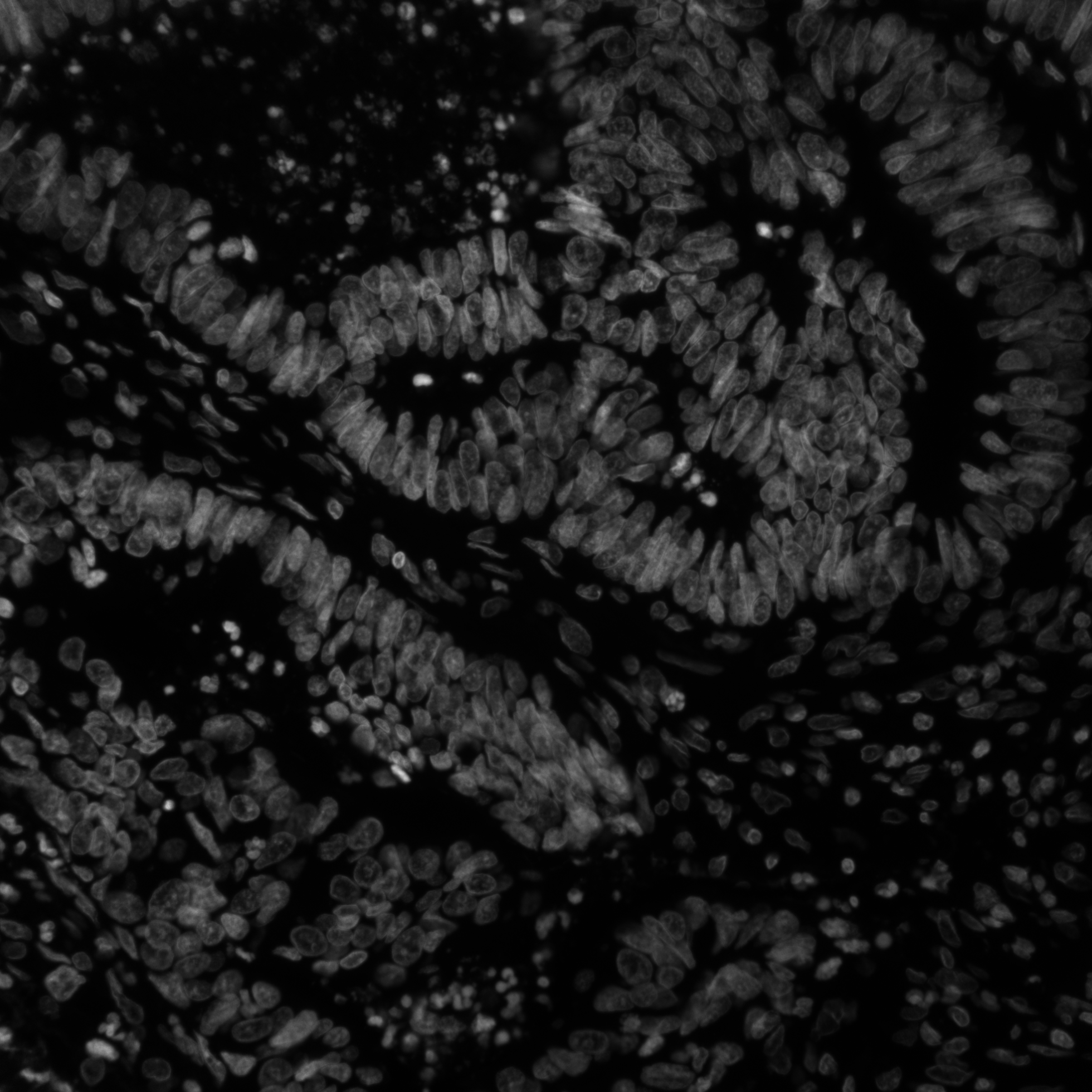

Supplement: Supplementary file 3 — Source data Fig. 1 [file 44318_2025_542_MOESM3_ESM.zip › Figure 1/1F/Ec_DAPI.tif]

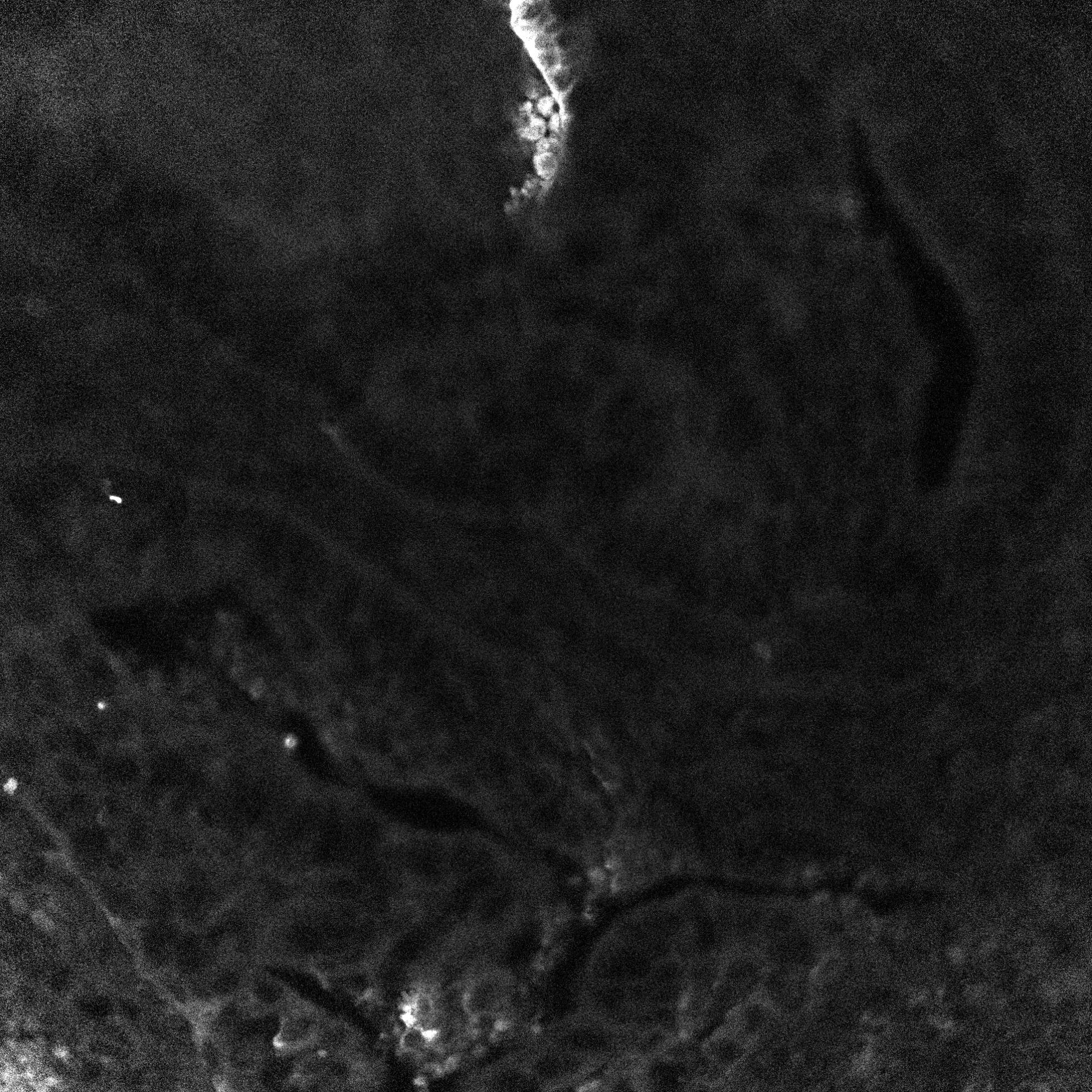

Supplement: Supplementary file 3 — Source data Fig. 1 [file 44318_2025_542_MOESM3_ESM.zip › Figure 1/1F/Ec_CY5.tif]

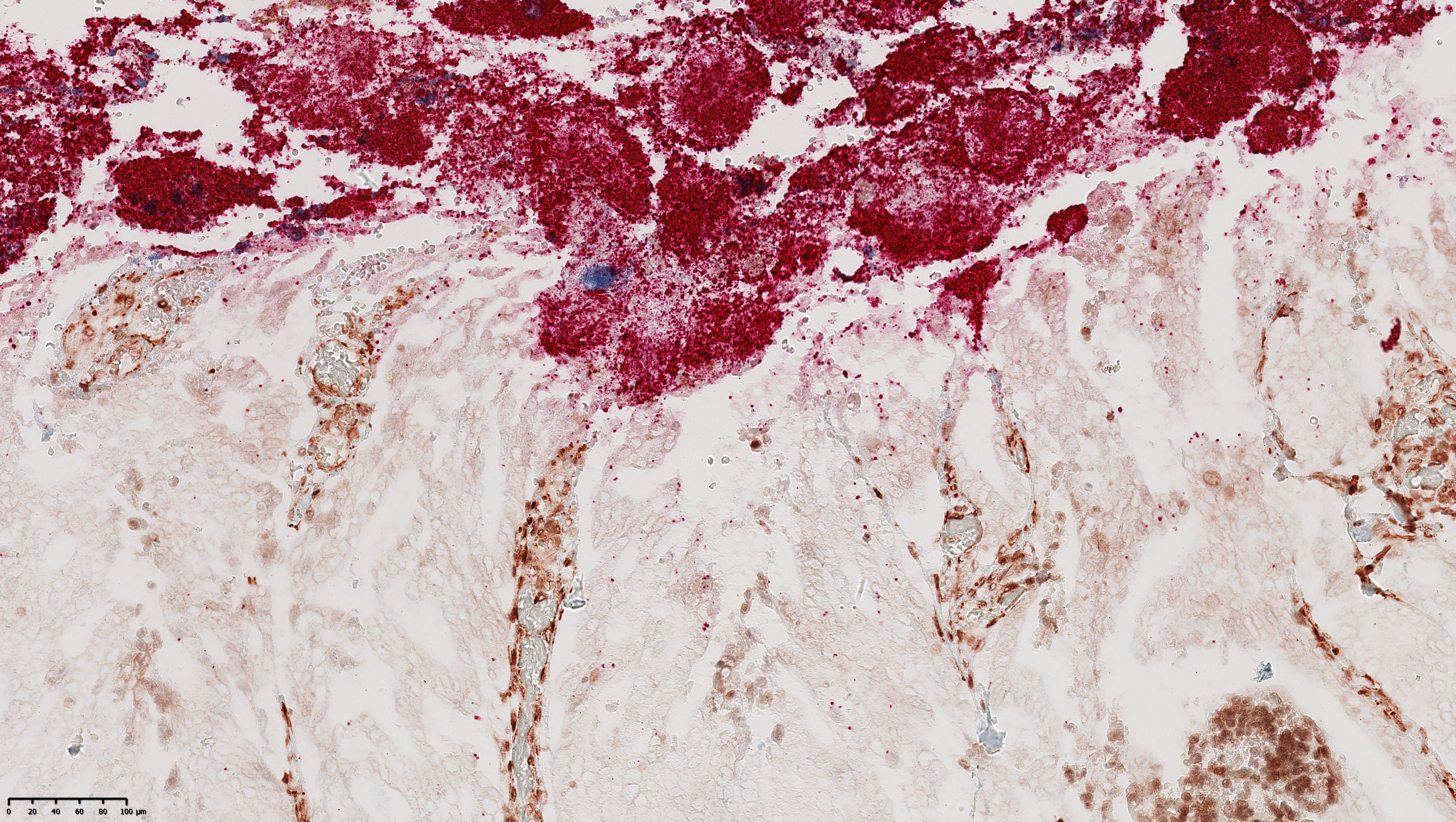

Supplement: Supplementary file 3 — Source data Fig. 1 [file 44318_2025_542_MOESM3_ESM.zip › Figure 1/1B/N23_100_03_SMA_8-120A7_8-120B1_cropped-tiff.tif]

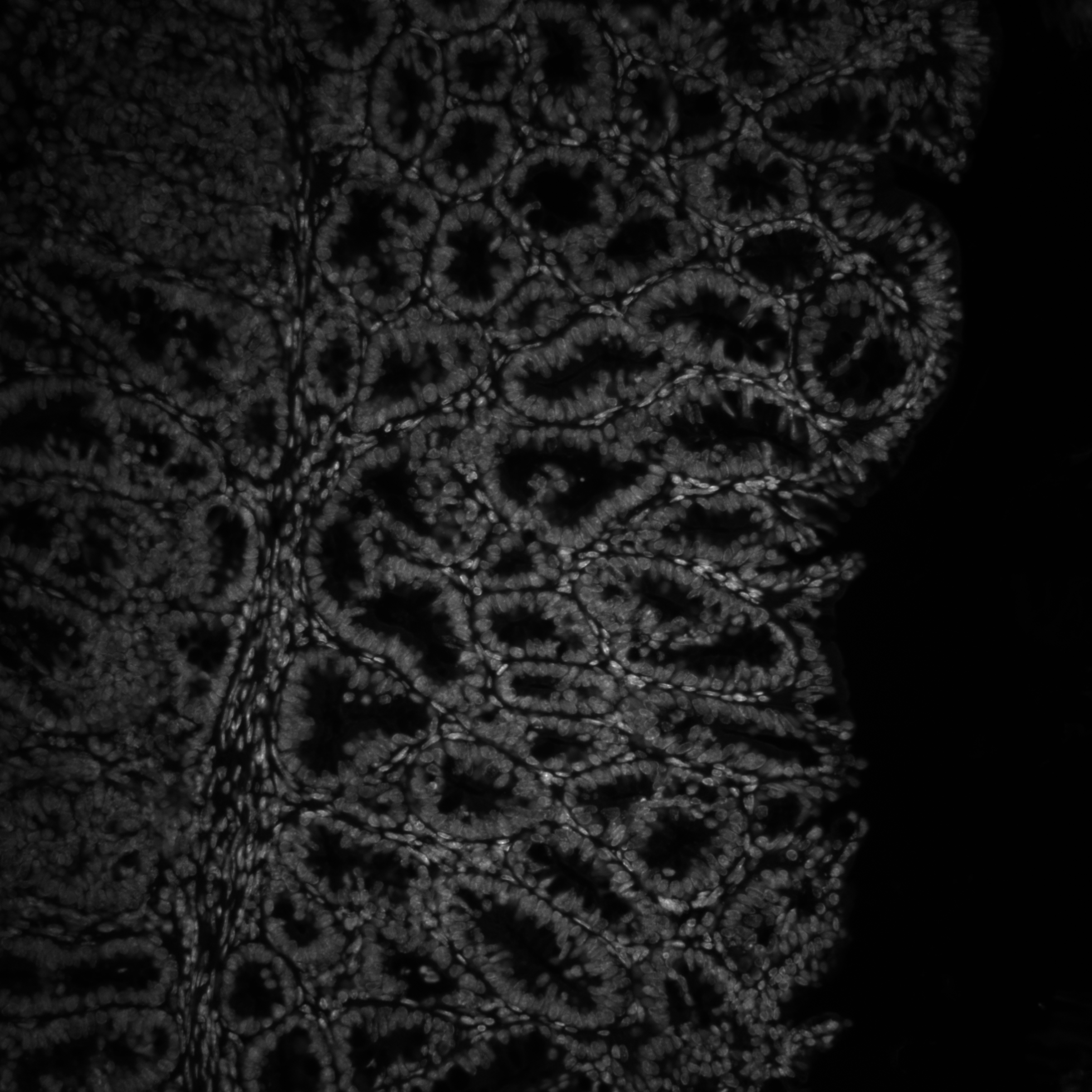

Supplement: Supplementary file 3 — Source data Fig. 1 [file 44318_2025_542_MOESM3_ESM.zip › Figure 1/1E/GF-CDX_colon_DAPI-PDPN-OMP.tif]

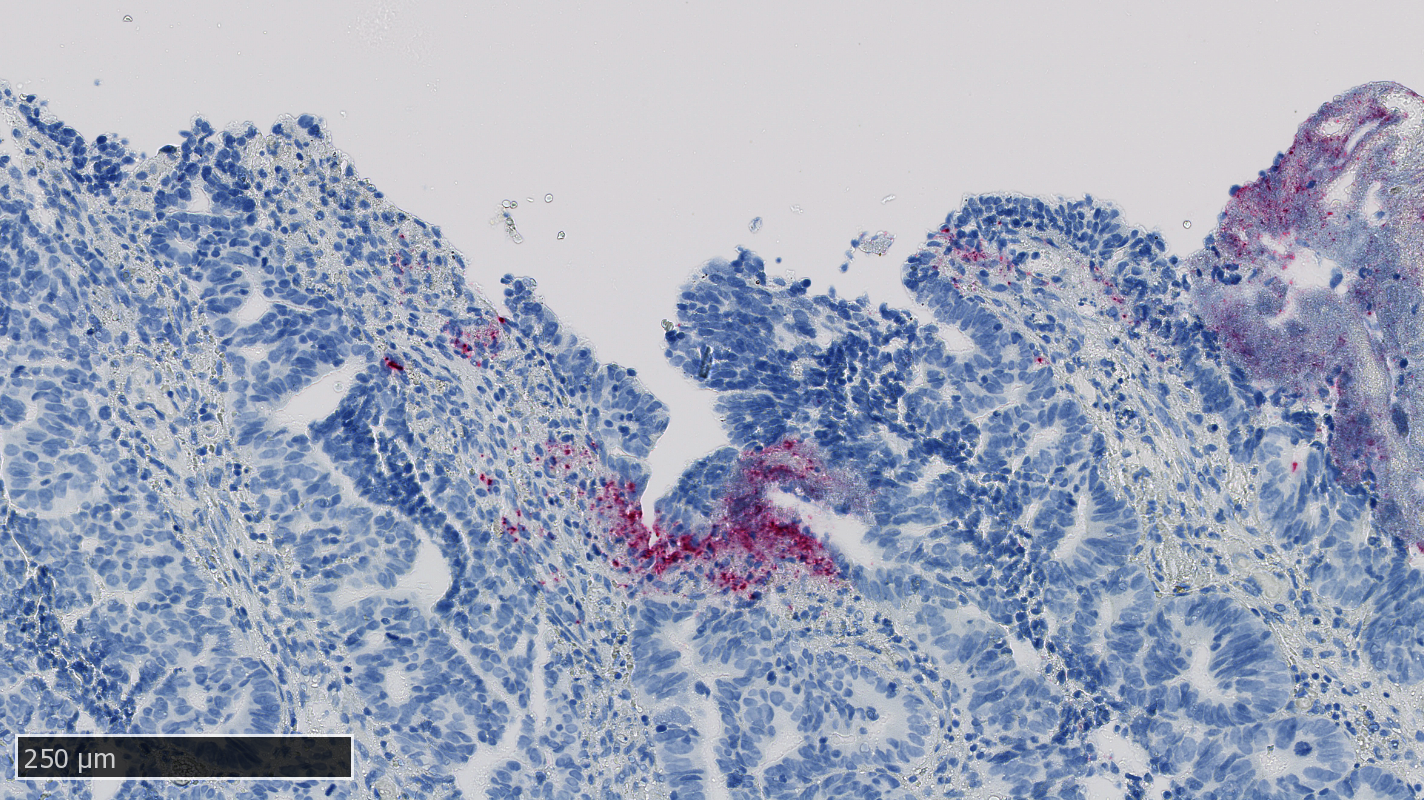

Supplement: Supplementary file 3 — Source data Fig. 1 [file 44318_2025_542_MOESM3_ESM.zip › Figure 1/1C/N25_015_01_FUSO_8-106A1+C24-07.8+C24-76.1_cropped-tiff.tif]

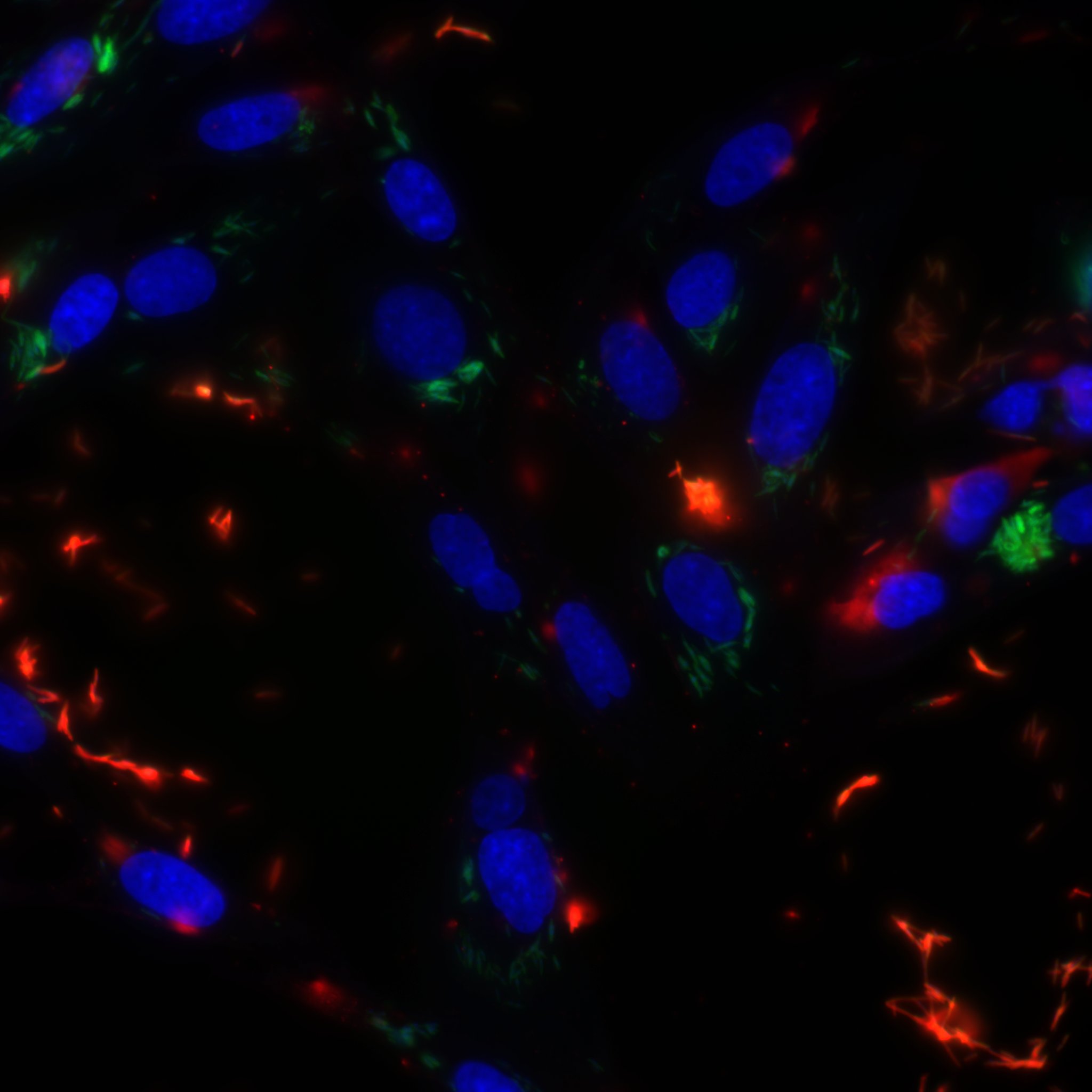

Supplement: Supplementary file 3 — Source data Fig. 1 [file 44318_2025_542_MOESM3_ESM.zip › Figure 1/1G/CT5.3/CT5.3_25586_S2_6_GOOD_merged.jpg]

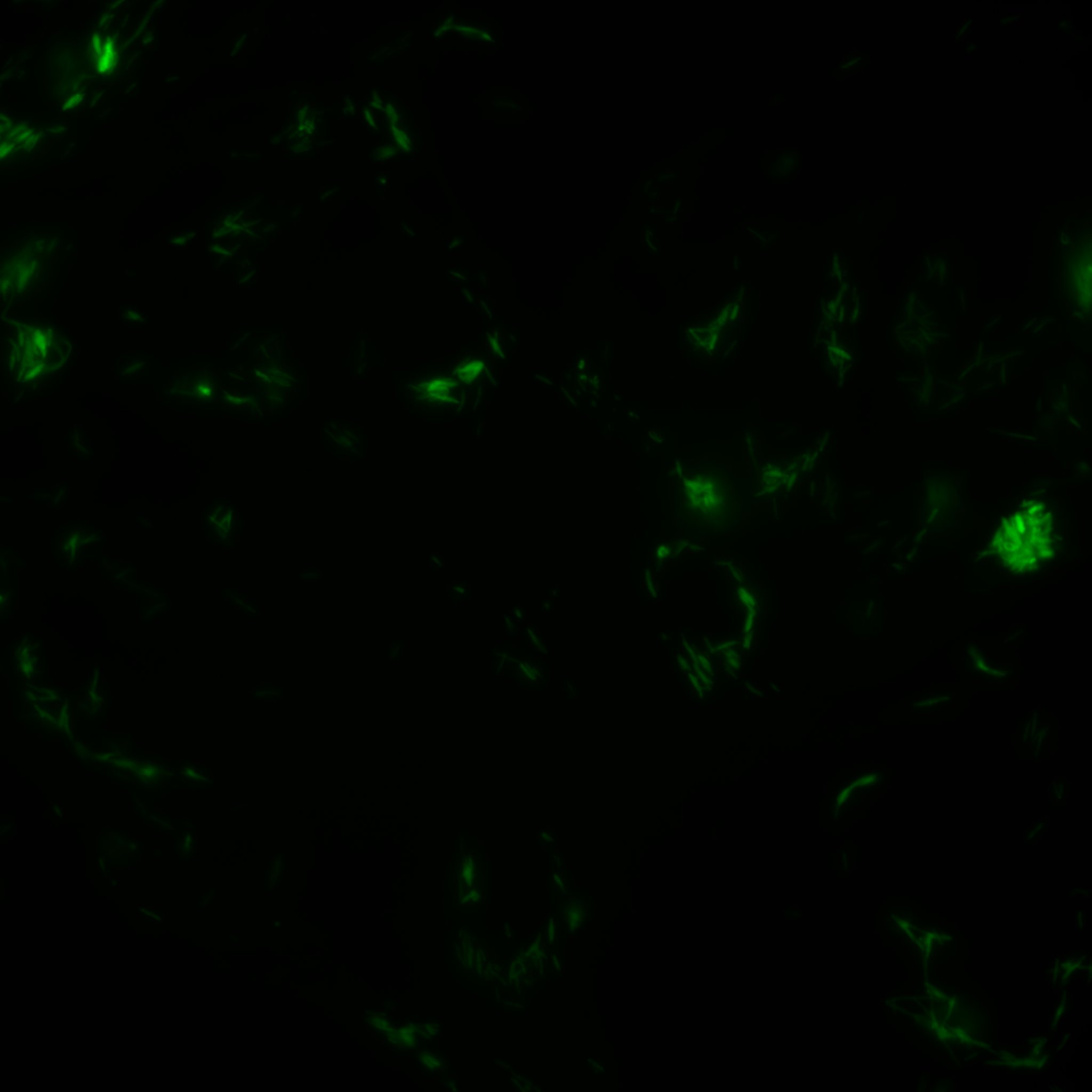

Supplement: Supplementary file 3 — Source data Fig. 1 [file 44318_2025_542_MOESM3_ESM.zip › Figure 1/1G/CT5.3/CT5.3_25586_S2_6_GOOD_green.jpg]

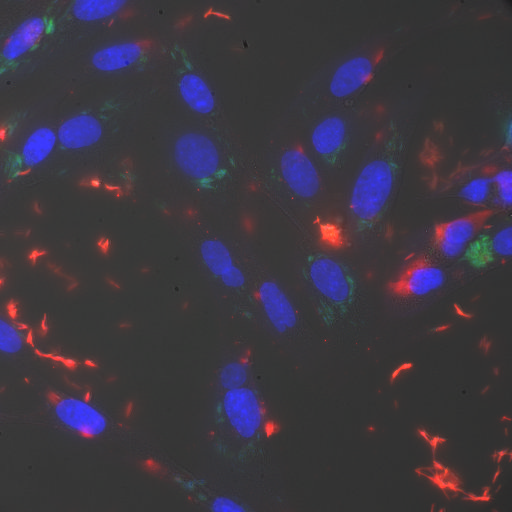

Supplement: Supplementary file 3 — Source data Fig. 1 [file 44318_2025_542_MOESM3_ESM.zip › Figure 1/1G/CT5.3/CT5.3_25586_S2_6_GOOD.vsi]

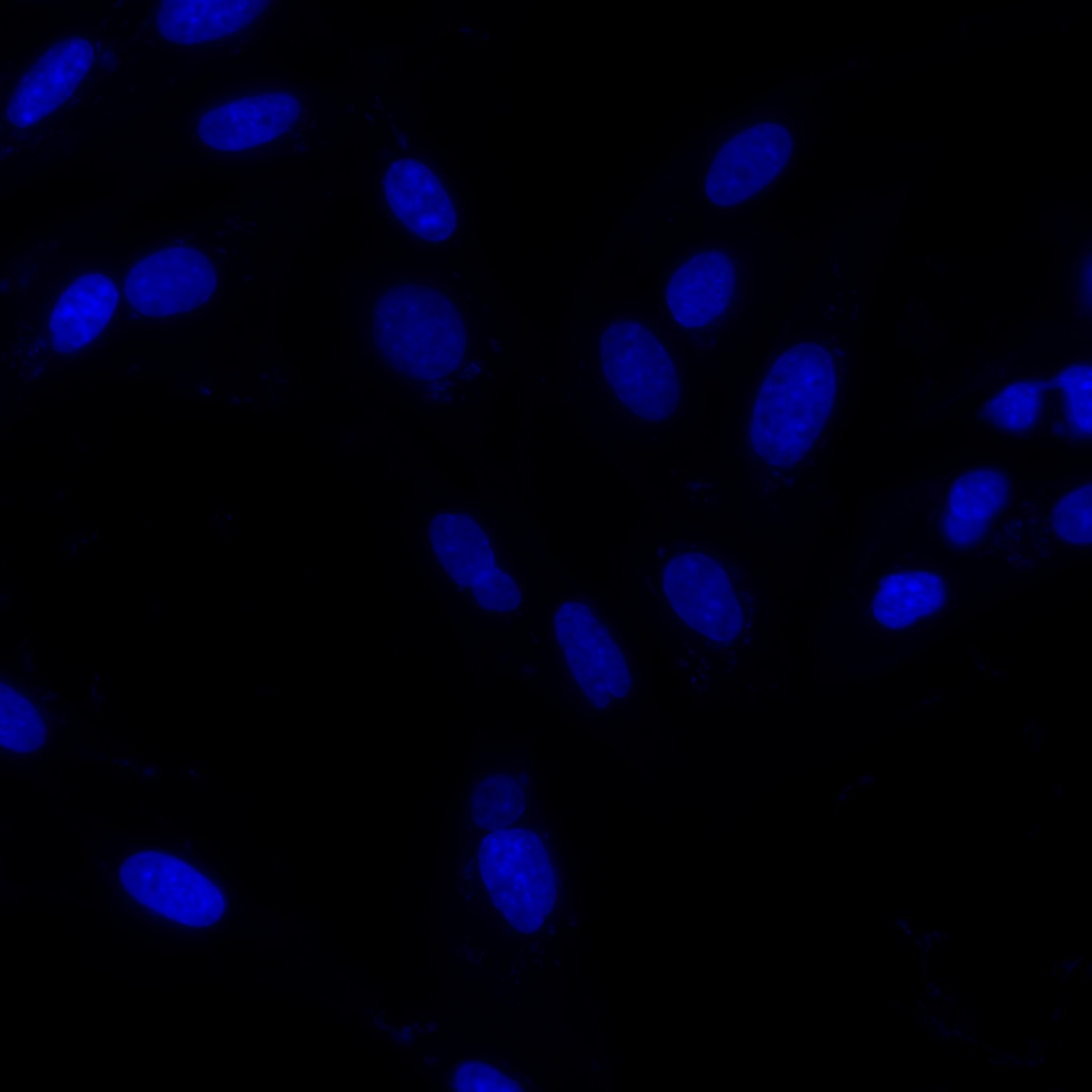

Supplement: Supplementary file 3 — Source data Fig. 1 [file 44318_2025_542_MOESM3_ESM.zip › Figure 1/1G/CT5.3/CT5.3_25586_S2_6_GOOD_blue.jpg]

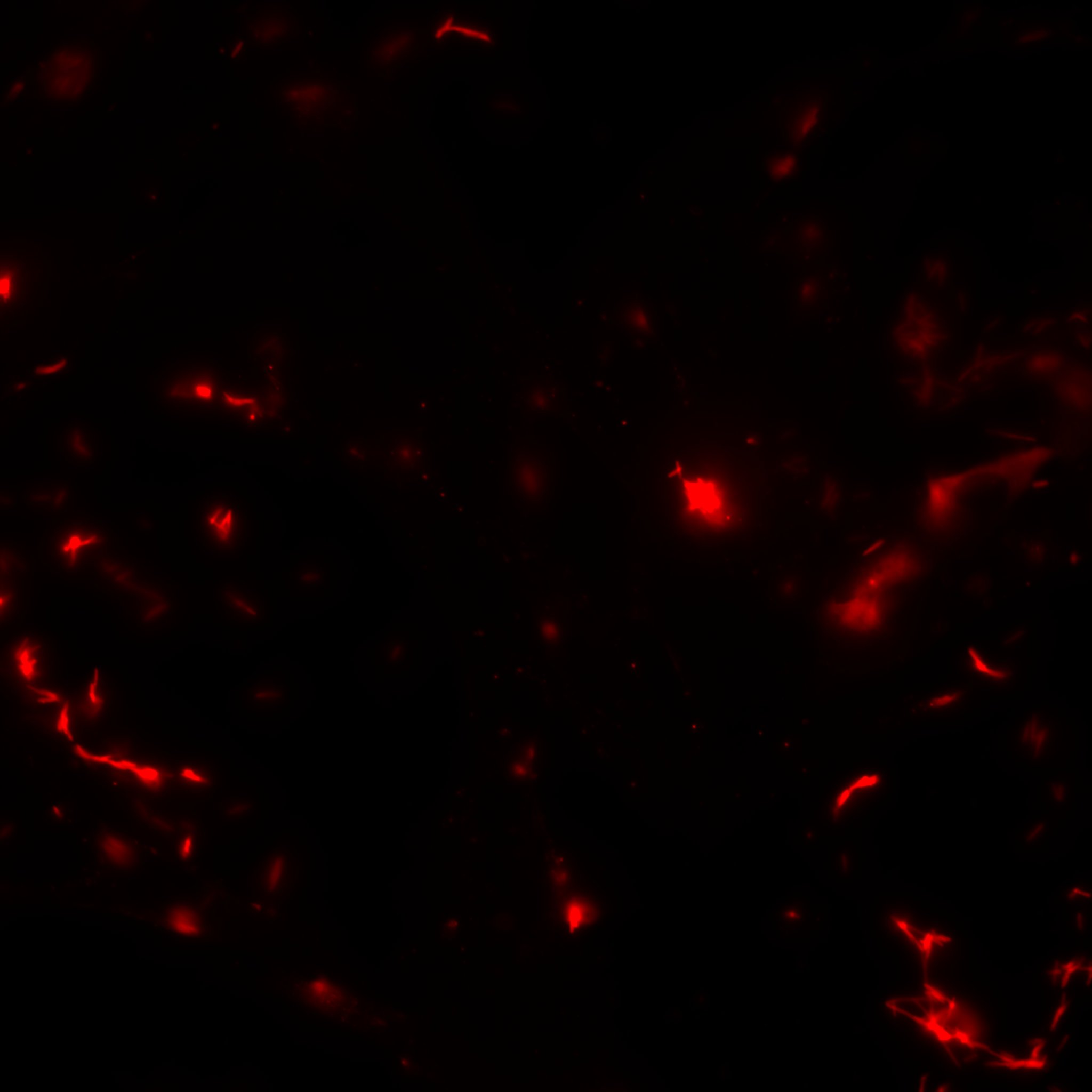

Supplement: Supplementary file 3 — Source data Fig. 1 [file 44318_2025_542_MOESM3_ESM.zip › Figure 1/1G/CT5.3/CT5.3_25586_S2_6_GOOD_red.jpg]

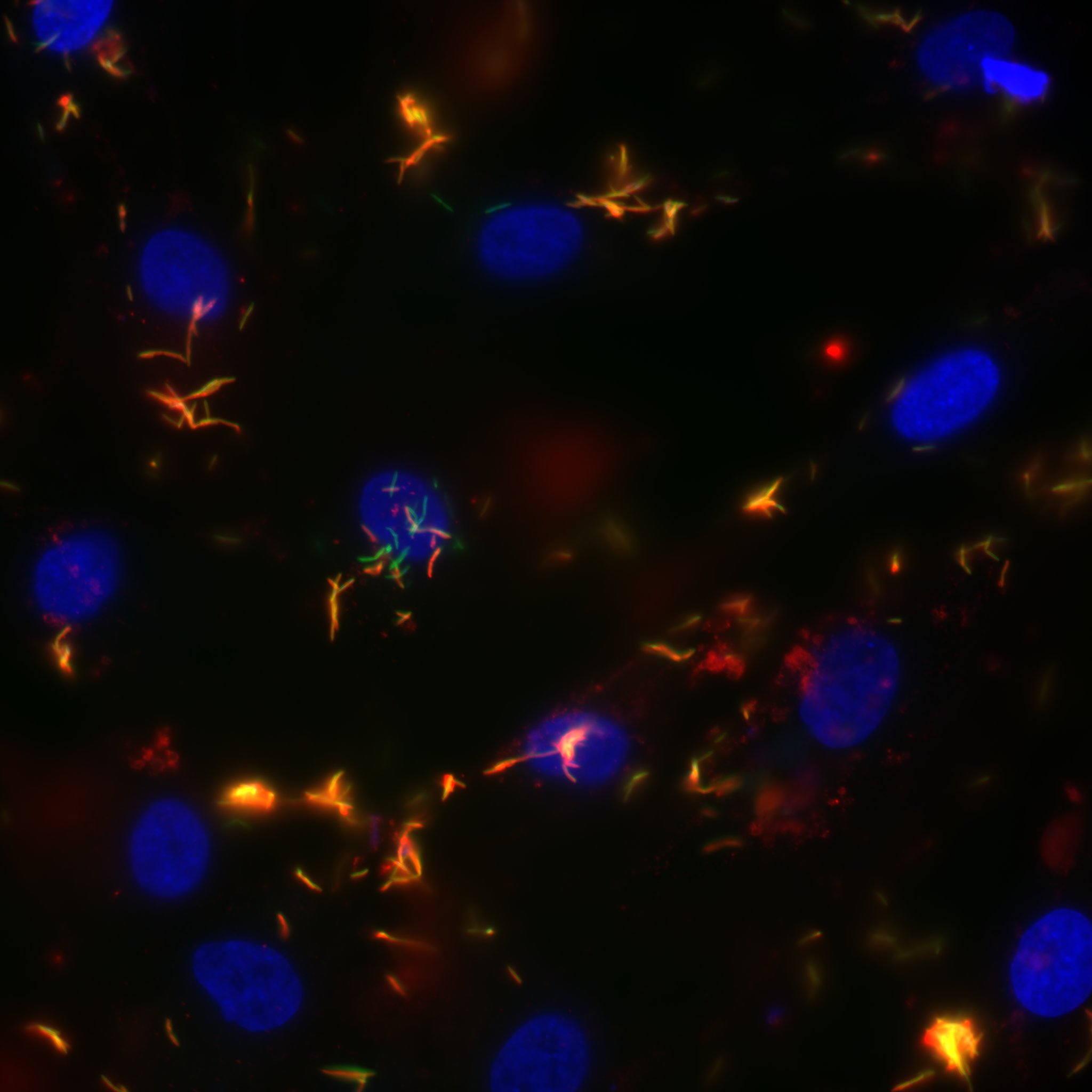

Supplement: Supplementary file 3 — Source data Fig. 1 [file 44318_2025_542_MOESM3_ESM.zip › Figure 1/1G/CAF05/CAF05_delta galkt-treated_good_1_merged.jpg]

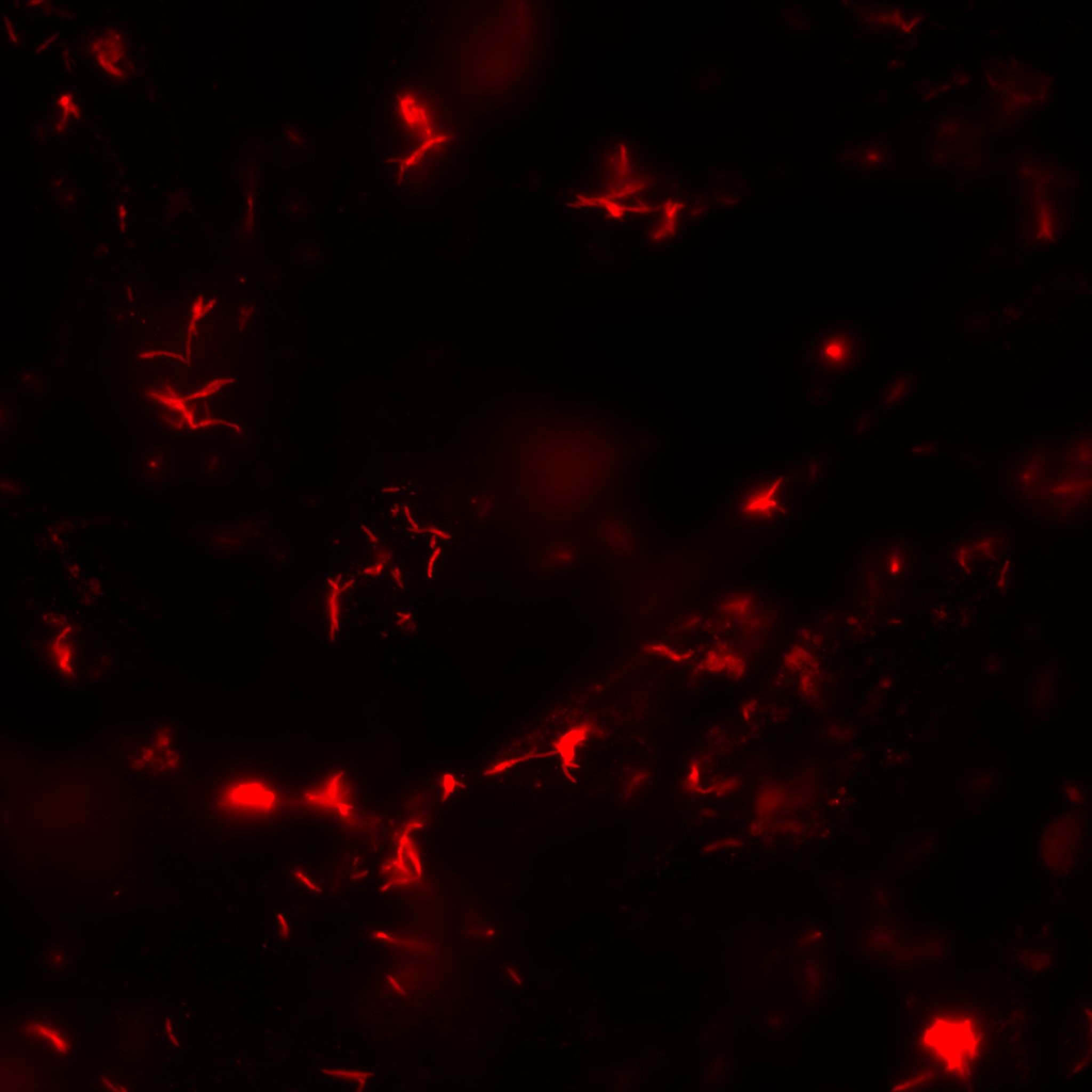

Supplement: Supplementary file 3 — Source data Fig. 1 [file 44318_2025_542_MOESM3_ESM.zip › Figure 1/1G/CAF05/CAF05_delta galkt-treated_good_1_red.jpg]

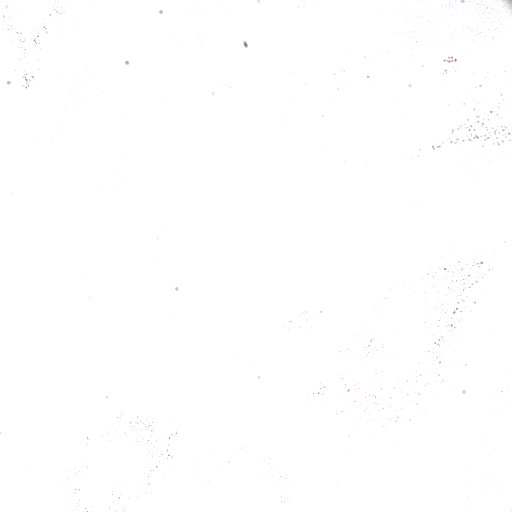

Supplement: Supplementary file 3 — Source data Fig. 1 [file 44318_2025_542_MOESM3_ESM.zip › Figure 1/1G/CAF05/CAF05_delta galkt-treated_good_1.vsi]

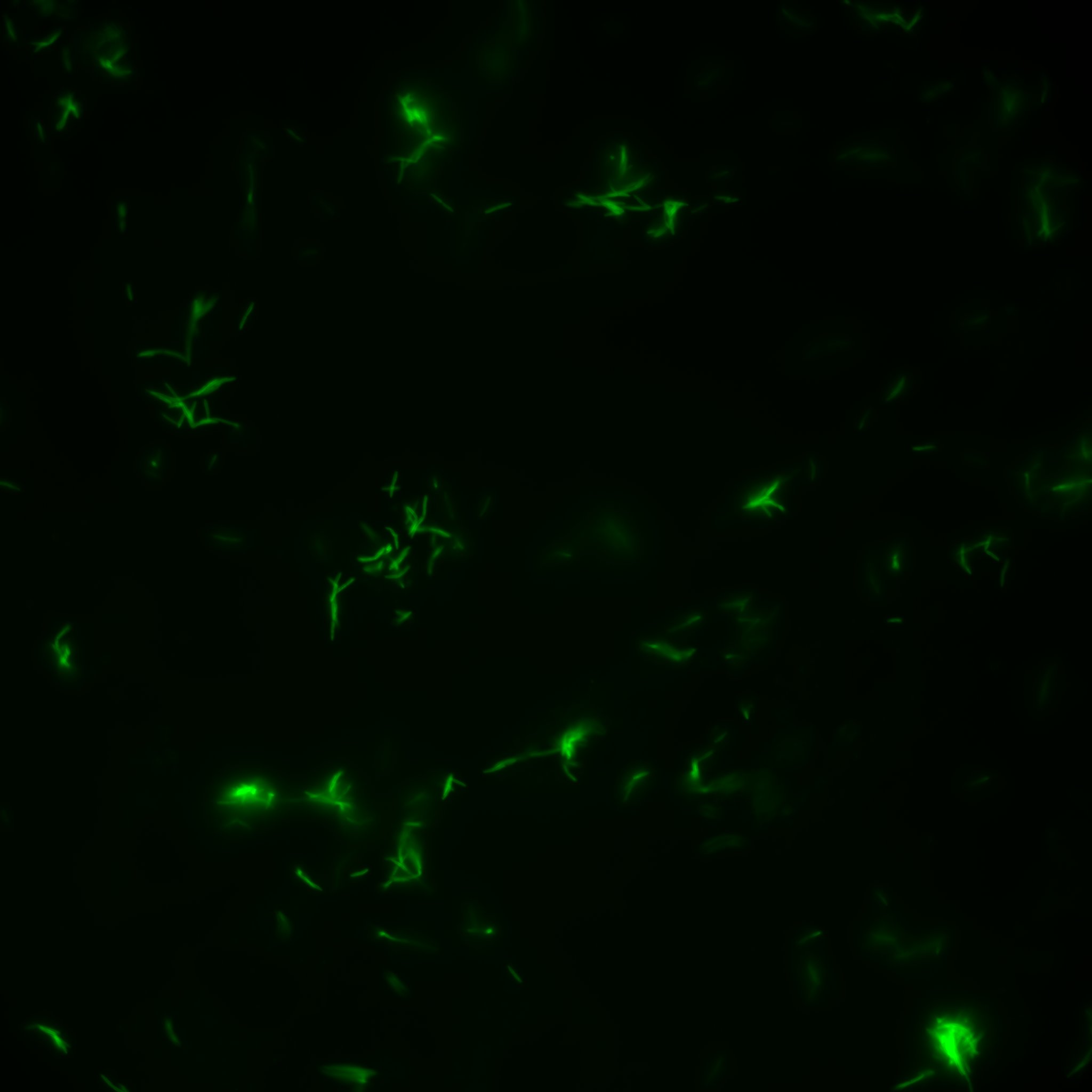

Supplement: Supplementary file 3 — Source data Fig. 1 [file 44318_2025_542_MOESM3_ESM.zip › Figure 1/1G/CAF05/CAF05_delta galkt-treated_good_1_green.jpg]

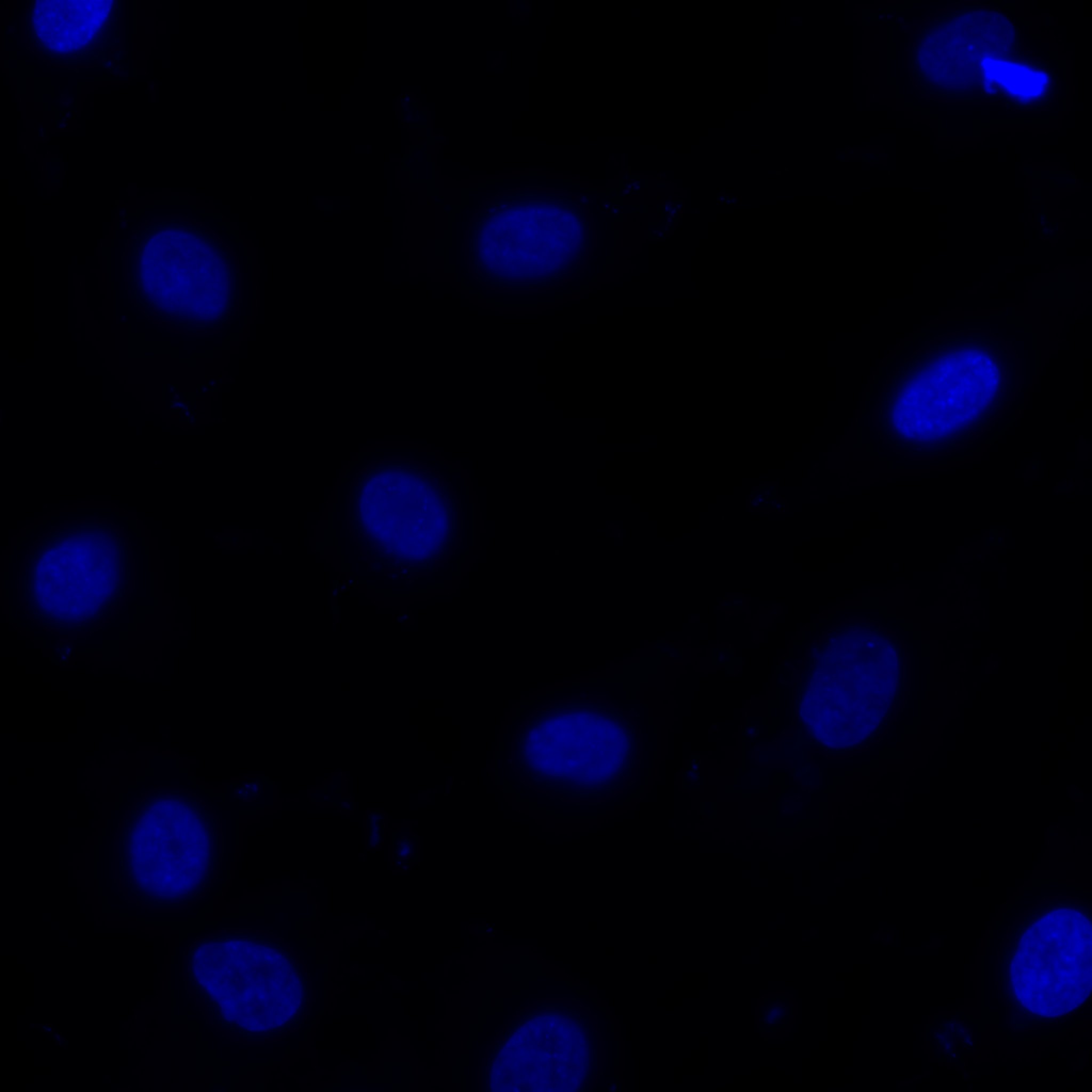

Supplement: Supplementary file 3 — Source data Fig. 1 [file 44318_2025_542_MOESM3_ESM.zip › Figure 1/1G/CAF05/CAF05_delta galkt-treated_good_1_blue.jpg]

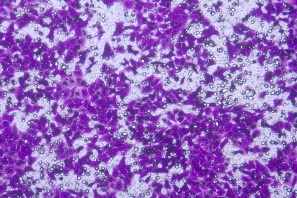

Supplement: Supplementary file 7 — Source data Fig. 5 [file 44318_2025_542_MOESM7_ESM.zip › Figure 5/5F/caf05_caf.jpeg]

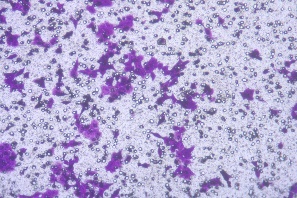

Supplement: Supplementary file 7 — Source data Fig. 5 [file 44318_2025_542_MOESM7_ESM.zip › Figure 5/5F/ct53_ctrl.jpeg]

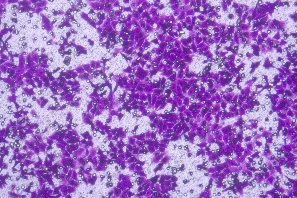

Supplement: Supplementary file 7 — Source data Fig. 5 [file 44318_2025_542_MOESM7_ESM.zip › Figure 5/5F/caf05_ec-500.jpeg]

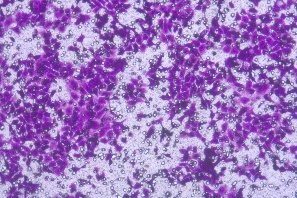

Supplement: Supplementary file 7 — Source data Fig. 5 [file 44318_2025_542_MOESM7_ESM.zip › Figure 5/5F/ct53_ec-500.jpeg]

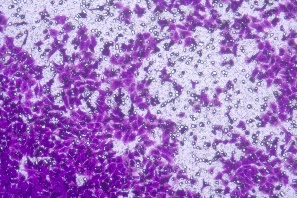

Supplement: Supplementary file 7 — Source data Fig. 5 [file 44318_2025_542_MOESM7_ESM.zip › Figure 5/5F/ct53_fn-50.jpeg]

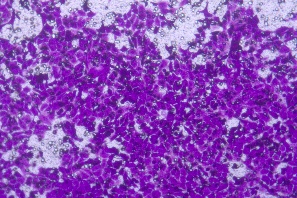

Supplement: Supplementary file 7 — Source data Fig. 5 [file 44318_2025_542_MOESM7_ESM.zip › Figure 5/5F/ct53_fn-500.jpeg]

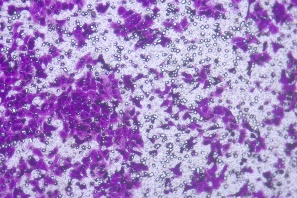

Supplement: Supplementary file 7 — Source data Fig. 5 [file 44318_2025_542_MOESM7_ESM.zip › Figure 5/5F/ct53_caf.jpeg]

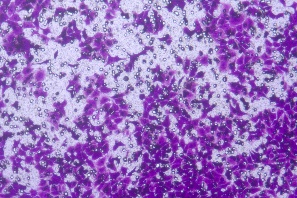

Supplement: Supplementary file 7 — Source data Fig. 5 [file 44318_2025_542_MOESM7_ESM.zip › Figure 5/5F/caf05_fn-50.jpeg]

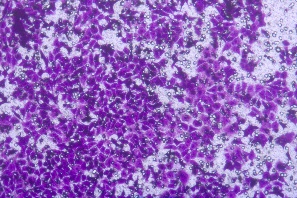

Supplement: Supplementary file 7 — Source data Fig. 5 [file 44318_2025_542_MOESM7_ESM.zip › Figure 5/5F/caf05_fn-500.jpeg]

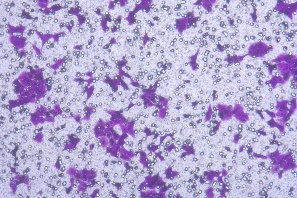

Supplement: Supplementary file 7 — Source data Fig. 5 [file 44318_2025_542_MOESM7_ESM.zip › Figure 5/5F/caf05_ctrl.jpeg]

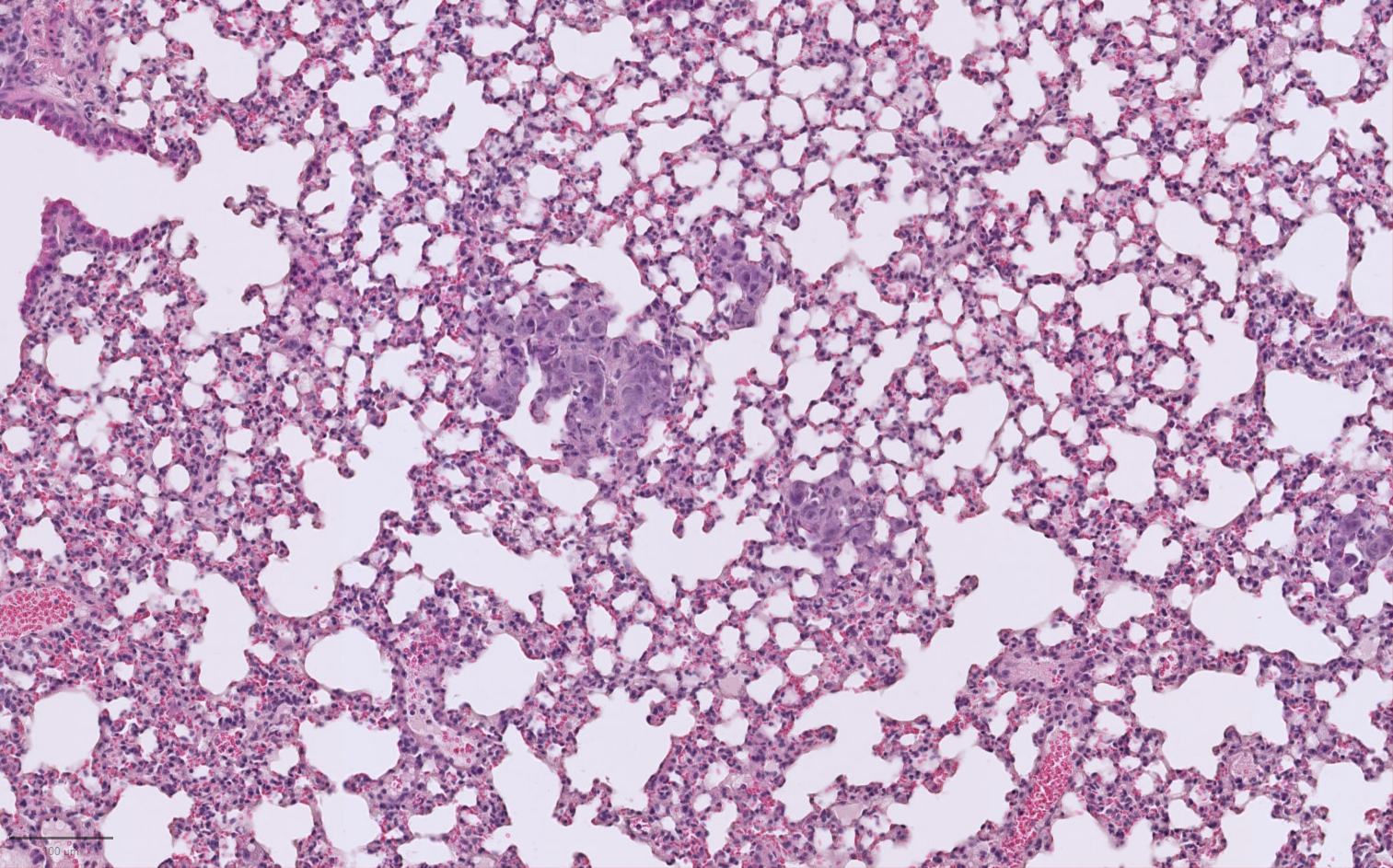

Supplement: Supplementary file 8 — Source data Fig. 6 [file 44318_2025_542_MOESM8_ESM.zip › Figure 6/6C/Ctrl_245.tif]

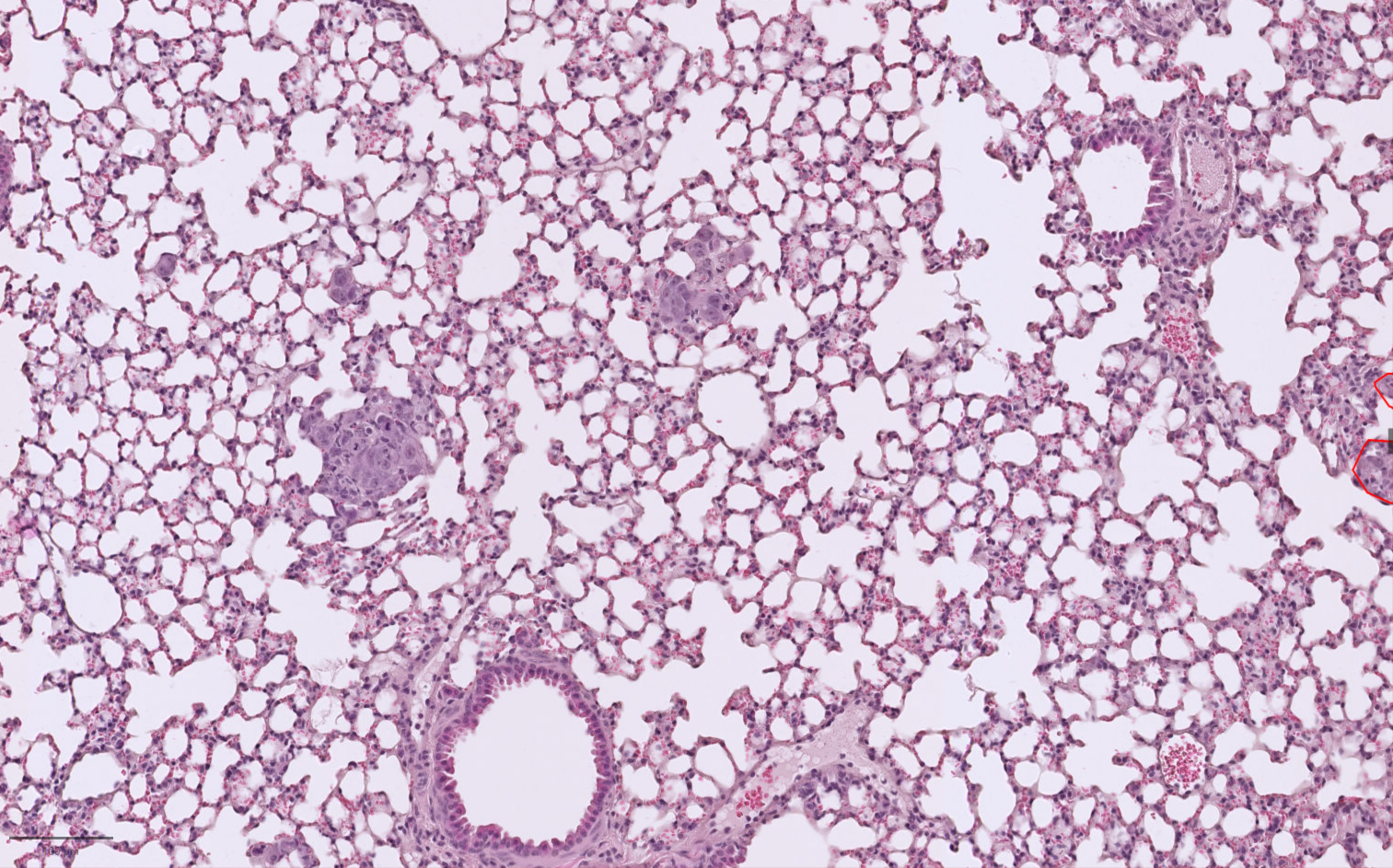

Supplement: Supplementary file 8 — Source data Fig. 6 [file 44318_2025_542_MOESM8_ESM.zip › Figure 6/6C/Ec_265.tif]

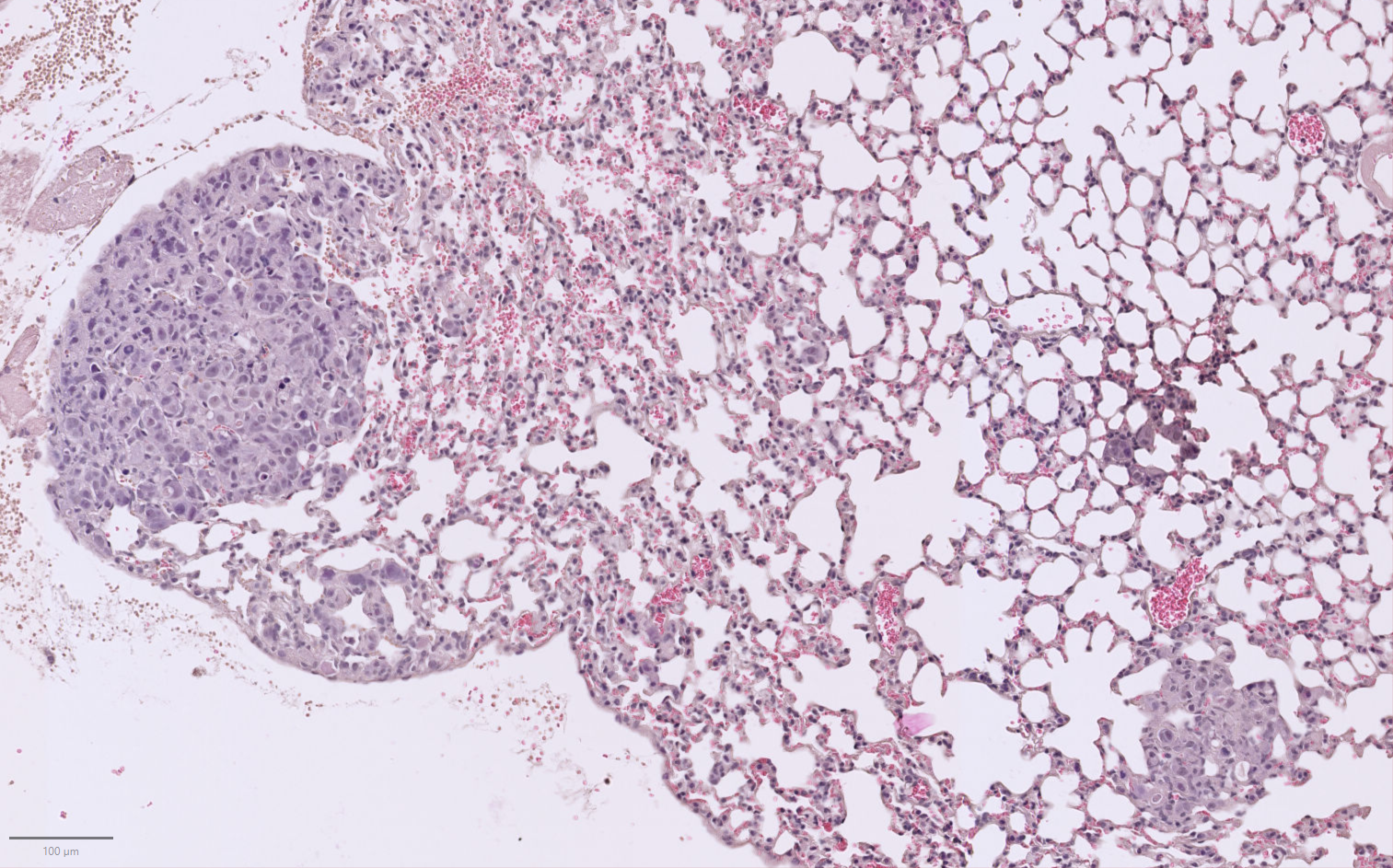

Supplement: Supplementary file 8 — Source data Fig. 6 [file 44318_2025_542_MOESM8_ESM.zip › Figure 6/6C/Fn_277.tif]
